# Discovery of an energy-dependent many-body process in the *K*β spectrum of manganese metal using extended-range high-energy-resolution fluorescence detection with advanced structural insights from principal component analysis

**DOI:** 10.1107/S2052252525005573

**Published:** 2025-08-21

**Authors:** Jack Stephens, Ramesh Rijal, Daniel Sier, Nicholas T. T. Tran, Jonathan W. Dean, Paul Di Pasquale, Tony Kirk, Minh Dao, Chanh Q. Tran, Shusaku Hayama, Sofia Diaz-Moreno, Christopher T. Chantler

**Affiliations:** a School of Physics University of Melbourne Melbourne Victoria Australia; b Department of Chemistry and Physics, La Trobe University, Melbourne, Victoria, Australia; c Diamond Light Source, Harwell Science and Innovation Campus Didcot OX11 0DE UK; Australian Nuclear Science and Technology Organisation and University of Wollongong, Australia

**Keywords:** extended-range high-energy-resolution fluorescence detection, materials science, inelastic X-ray scattering, X-ray absorption fine structure, manganese, *K*β spectra, shake processes, principal component analysis, satellites, many-body reduction factor, many-body processes, X-ray emission spectroscopy

## Abstract

The discovery of the novel *n* = 2 satellite transition in the *K*β emission spectrum of manganese and its evolution with incident photon energy are presented. Using the XR-HERFD (extended-range high-energy-resolution fluorescence detection) technique, we conclusively demonstrate the existence of this phenomenon with a statistical significance corresponding to 652 σ_se_ across the measured spectrum, far above the discovery threshold of 3–6 σ_se_. We apply principal component analysis (PCA) to the XR-HERFD data to extract advanced structural insights. The evolution of this novel spectral feature and physical process are quantified by incorporating regression, revealing the increase in intensity over a wide range of incident photon energies. We validate these findings through independent test data. These results directly challenge the conventional treatment of the many-body reduction factor *S*_0_^2^ as a constant independent of incident photon energy in the standard XAFS (X-ray absorption fine structure) equation. Thereby, these results present compelling evidence that *S*_0_^2^ should be modelled as a varying function of incident photon energy, marking the first observation of this behaviour in *K*β spectra. This facilitates a greater quantitative understanding of HERFD spectra and a comprehensive representation of many-body effects in condensed matter systems.

## Abstract 2

A new satellite is discovered in the manganese *K*β spectrum using extended-range high-energy-resolution fluorescence detection. Advanced insights on its structure and evolution are extracted with principal component analysis.

## 1. Introduction

Core–hole spectroscopy describes the measurement of fluorescent photons emitted during the decay of a hole state created by the photoionization of a 1*s* electron (Glatzel *et al.*, 2001 ▸; de Groot & Kotani, 2008 ▸; Ament *et al.*, 2011 ▸; Glatzel *et al.*, 2013 ▸). Many applications of core–hole spectroscopy centre around *K*β transitions, which arise when a 3*p* electron fills the core vacancy. The 3*d* transition metals, such as manganese, garner a great deal of contemporary interest in *K*β spectroscopy due to their strong coupling between unpaired electrons in the 3*d* subshell and the 3*p* hole state which gives rise to distinctive fine structure (Gamblin & Urch, 2001 ▸; Glatzel *et al.*, 2003 ▸; Glatzel & Bergmann, 2005 ▸; Beckwith *et al.*, 2011 ▸; Bauer, 2014 ▸). As a result, recent applications of X-ray emission spectroscopy (XES) have employed *K*β transitions to investigate structure, spin state and oxidation number in transition metals, leading to applications in materials science (Vankó *et al.*, 2006 ▸; Lafuerza *et al.*, 2020 ▸) and bioinorganic chemistry (Pollock & DeBeer, 2011 ▸; Rees *et al.*, 2016 ▸; Martin-Diaconescu *et al.*, 2016 ▸) and investigations of the reaction mechanisms of catalysts and batteries *in operando* (Lancaster *et al.*, 2011 ▸; Günter *et al.*, 2016 ▸).

Recent research efforts in core–hole spectroscopy centre on many-body phenomena, now referred to as shake processes (Millikan, 1917 ▸; Åberg, 1967 ▸). The study of shake-off events, wherein two electrons are ejected simultaneously from an atomic system following interaction with a singular incident photon, are a particular recent area of theoretical and experimental investigation (Kheifets, 2022 ▸; Pedersen *et al.*, 2023 ▸; Kavčič & Žitnik, 2024 ▸; Sier *et al.*, 2024 ▸; Dean *et al.*, 2024 ▸). Fluorescence transitions following shake-off events are referred to as satellites. Due to the presence of an additional electron hole, the characteristic fluorescent photon emitted in a satellite transition is non-degenerate to the single-body diagram counterpart, and hence satellite transitions can explain high emission energy (*E*_em_) peaks and asymmetries in X-ray emission spectra (Hölzer *et al.*, 1997 ▸).

Shake-off events have a significant impact on the X-ray absorption fine structure (XAFS). The fine structure in absorption spectra reveals crucial structural information, including the type and location of constituent atoms (Stern, 1978 ▸). In modern condensed-matter physics, it is modelled by an equation of the form (Rehr & Albers, 2000 ▸)
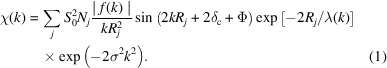
The meaning of these parameters is discussed in great detail elsewhere (Stern, 1974 ▸; Lee & Pendry, 1975 ▸; Rehr *et al.*, 1978 ▸; Rehr & Albers, 2000 ▸; Gaur *et al.*, 2013 ▸; Chantler *et al.*, 2024 ▸). Most important to this work is 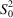, which represents an overlap (damping) factor that quantifies how the atomic potential experienced by passive electrons changes between the initial and final states due to the photoionization of a core electron, quantifying the probability that only a single photoelectron is ejected (Lee & Beni, 1977 ▸; Rehr *et al.*, 1978 ▸; Roy *et al.*, 2001 ▸). Physically, 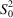 is intended to represent a reduction in the amplitude of XAFS oscillations due to many-body phenomena, and is thus referred to as the many-body reduction factor.

In the literature 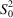 is typically assigned a constant value between 0.6 and 0.9 (Rehr *et al.*, 1978 ▸), derived from the total proportion of many-body events in the limit of high photon energy (the sudden approximation), or unity. However, this assumption often results in suboptimal fits to experimental data, particularly in transition metals (Roy *et al.*, 2001 ▸). Experimental observations show that the proportion of many-body events increases with incident photon energy (*E*_inc_) beyond a certain threshold (a phenomenon we refer to henceforth as evolution) (Carlson & Krause, 1965 ▸; Stöhr *et al.*, 1983 ▸). This threshold, known as the onset, occurs when *E*_inc_ exceeds the sum of the core and shake electron binding energies and the satellite transition becomes energetically allowed. These findings have led to the proposal that 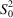 should be corrected to take some energy-dependent form (Lee & Beni, 1977 ▸; Tran *et al.*, 2023 ▸; Sier *et al.*, 2024 ▸). A comprehensive reformulation of 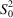 requires precise measurement of many-body processes in a given material. No studies to date have reported measurements of evolving shake-off satellites in *K*β spectra, highlighting a significant gap in the literature. To interrogate the nature of 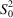 in the *K*β fluorescence channel, we employ the XR-HERFD technique to collect two-dimensional spectra over an extended range of *E*_inc_ and observe new many-body processes in the *K*β spectrum of manganese. By isolating prominent components of the data, we present the first quantified amplitude profile of such a process in a *K*β spectrum, showcasing quantum evolution.

## 2. The XR-HERFD technique

Measuring the variance in intensity of an atomic transition with incident photon energy requires the use of a two-dimensional spectroscopic technique such as RIXS (resonant inelastic X-ray scattering) that employs a combination of X-ray absorption spectroscopy (XAS) and X-ray emission spectroscopy (XES) (Eisenberger *et al.*, 1976 ▸; Guo *et al.*, 1995 ▸; Glatzel & Bergmann, 2005 ▸; Ament *et al.*, 2011 ▸; Schmitt *et al.*, 2014 ▸; Gel’mukhanov *et al.*, 2021 ▸). RIXS requires high-resolution detection apparatus and high-flux photon sources (Sier *et al.*, 2025 ▸), and so the advent of HERFD (high-energy-resolution fluorescence detection) spectroscopy in the late twentieth century has catalysed its experimental realization (Hämäläinen *et al.*, 1991 ▸). HERFD substantially improves experimental resolution by isolating a specific transmission line as a fluorescence channel, minimizing the effect of core–hole lifetime broadening on the achievable energy resolution of the fluorescence spectrum (Glatzel *et al.*, 2013 ▸; Günter *et al.*, 2016 ▸). Often, this allows for an energy resolution finer than the core–hole lifetime, which has facilitated a boom in core–hole spectroscopy (Hämäläinen *et al.*, 1991 ▸) and enabled detailed investigations into shake-off satellites (Deutsch *et al.*, 1996 ▸), which are often suppressed by several orders of magnitude relative to their single-body analogues (Dean *et al.*, 2024 ▸).

RIXS scans, which typically focus on resonant transitions in the pre-edge region (Eisenberger *et al.*, 1976 ▸; Glatzel & Bergmann, 2005 ▸; Ament *et al.*, 2011 ▸), are often insufficient to investigate shake-off events. To address this limitation, the XR-HERFD (extended-range HERFD) technique was developed to investigate the structure and evolution of novel processes well above a given transition edge (Tran *et al.*, 2023 ▸; Sier *et al.*, 2024 ▸; Sier *et al.*, 2025 ▸). In this work, the XR-HERFD technique is employed to uncover the structure and evolution of a previously undetected satellite transition in the *K*β spectrum of manganese metal.

## 3. Experimental measurement

**Figure 1:**
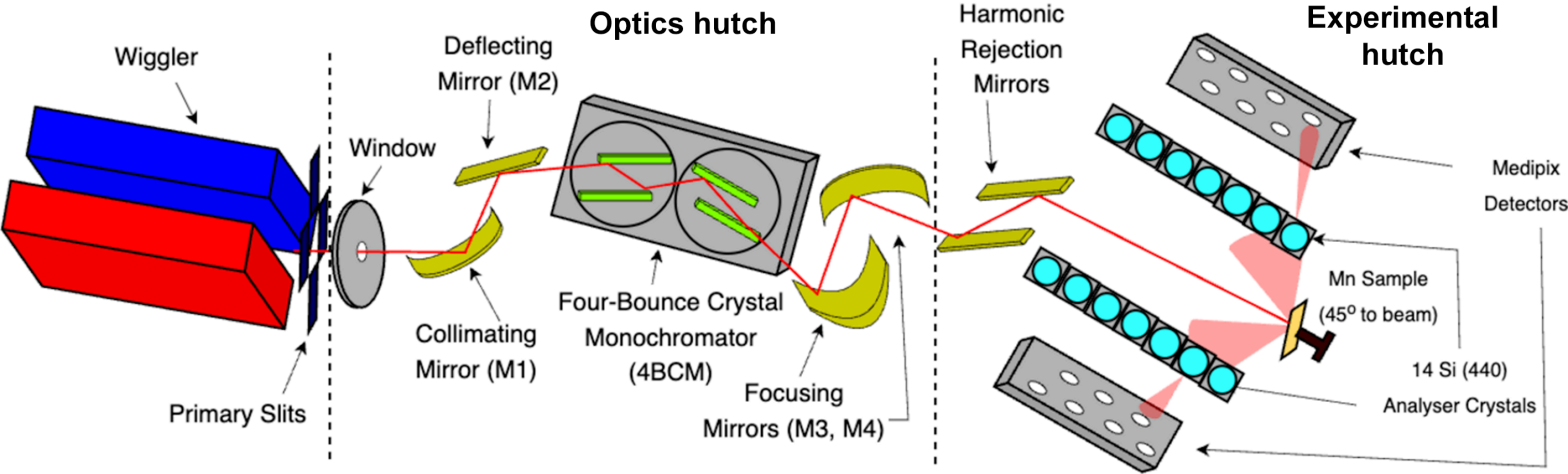
Schematic diagram of the beamline apparatus on I20-Scanning, which includes several advanced optical components. X-rays delivered by the wiggler source are deflected upwards by a vertical collimating mirror with rhodium and platinum stripes (M1) to maximize flux, before being downward deflected by the deflecting mirror (M2) to restore the beam’s horizontal trajectory and isolate any mirror adjustments. The X-rays then pass through the custom-built Si(111) four-bounce crystal monochromator (4BCM), ensuring the incident X-ray energy is well defined. Vertical and horizontal focusing mirrors (M3, M4) after the monochromator focus the X-rays on the sample position, while a set of harmonic rejection mirrors with rhodium and silicon stripes maintain spectral purity by reducing the content of higher-order harmonics in the X-ray beam before it is incident on the manganese sample. Fluorescent X-rays from the sample are Bragg-reflected by two sets of seven spherically bent and cylindrically sliced Si(440) analyser crystals, angled close to back-scattering. The analysers lie on a Rowland circle of 1 m diameter in the vertical plane, based on Johann-type geometry. Fluorescent photons are directed by the analysers to seven independent regions on each of the two respective Medipix detectors. This setup allows 14 separable and independent measurements of the XR-HERFD signal.

Following Tran *et al.* (2023 ▸) and Sier *et al.* (2024 ▸), measurements were conducted on the I20-Scanning beamline at Diamond Light Source. The experimental apparatus, depicted in Fig. 1 ▸, ensures high incident photon flux (∼ 10^12^ s^−1^) and beam uniformity and monochromaticity through a combination of focusing, collimating and harmonic rejection mirrors alongside a purpose-built four-bounce monochromator (Diaz-Moreno *et al.*, 2009 ▸; Diaz-Moreno, 2012 ▸; Hayama *et al.*, 2021 ▸; Sier *et al.*, 2025 ▸). Fourteen Si(440) Bragg analyser crystals in the Johann geometry were employed, which recorded the *K*β_1,3_ maximum at a Bragg angle θ_B_ ≃ 84.2°. This configuration significantly increases the total subtended detector solid angle relative to previous results in manganese *K*α that employed at most three Bragg analyser crystals, hypothetically improving the statistical quality by a factor of 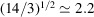.

Fluorescent photons of the allowed energy are Bragg-reflected by the analyser crystals onto two MAXIPIX (multichip area X-ray detector based on a photon-counting pixel array) TAA22PC detectors, an advanced iteration of the Medipix2 single-photon counting pixel detector. The detectors are configured in a 4×1 arrangement, creating a total pixel grid of 1024 × 256 pixels, and are collectively referred to as Medipix detectors for simplicity. Each analyser is arranged independently in the Johann geometry, lying on a Rowland circle with a diameter of 1 m with the sample and respective Medipix detector.

### 3.1. Data processing

The processing procedure for experimental data follows a methodology similar to that used in the analysis of manganese *K*α spectra (Sier *et al.*, 2025 ▸). A region of interest (RoI) is defined around the image of each analyser crystal on the Medipix detector to minimize false counts from electronic noise and stray background photons that are not Bragg-reflected by the analyser crystals. A corner of the detector region well removed from any RoI is selected to define inherent background counts at each incident energy, which were subtracted from the intensities recorded in each RoI. The resulting intensities were then normalized by upstream ion counts.

The next processing step, known as binary data splicing, addresses variations in the Bragg angle θ_B_ seen in the dispersion direction of the detector apparatus, corresponding to the vertical plane of the analyser crystals. These variations arise as the Bragg condition is precisely met only at the centre of each analyser crystal, due to their finite size and the geometry of the detector apparatus (Moretti Sala *et al.*, 2018 ▸). To correct for this, the RoI of each analyser crystal was divided into ten narrow horizontal slices. A Gaussian profile is then fitted to the peak of the *K*β_1,3_ transition of the isolated emission spectrum of each slice, aligning them to a reference spectrum.

Following alignment, the fluorescent intensities are extracted for each analyser crystal by aggregating across the ten slices. A sum is performed across each of the analyser crystals, weighted by standard error counts, to obtain the final processed XR-HERFD data. By incorporating binary data splicing, intrinsic instrumental broadening is minimized, increasing the limiting resolution of the data by approximately 10%, resulting in a fluorescent energy bandwidth of 0.61 eV. As a result, any remaining broadening in the XR-HERFD spectrum can be attributed predominantly to physical phenomena and investigated by advanced theory. A quantified description of the improvements afforded by an application of binary data splicing is given by Sier *et al.* (2025 ▸) and Rijal *et al.* (2025 ▸).

**Figure 2:**
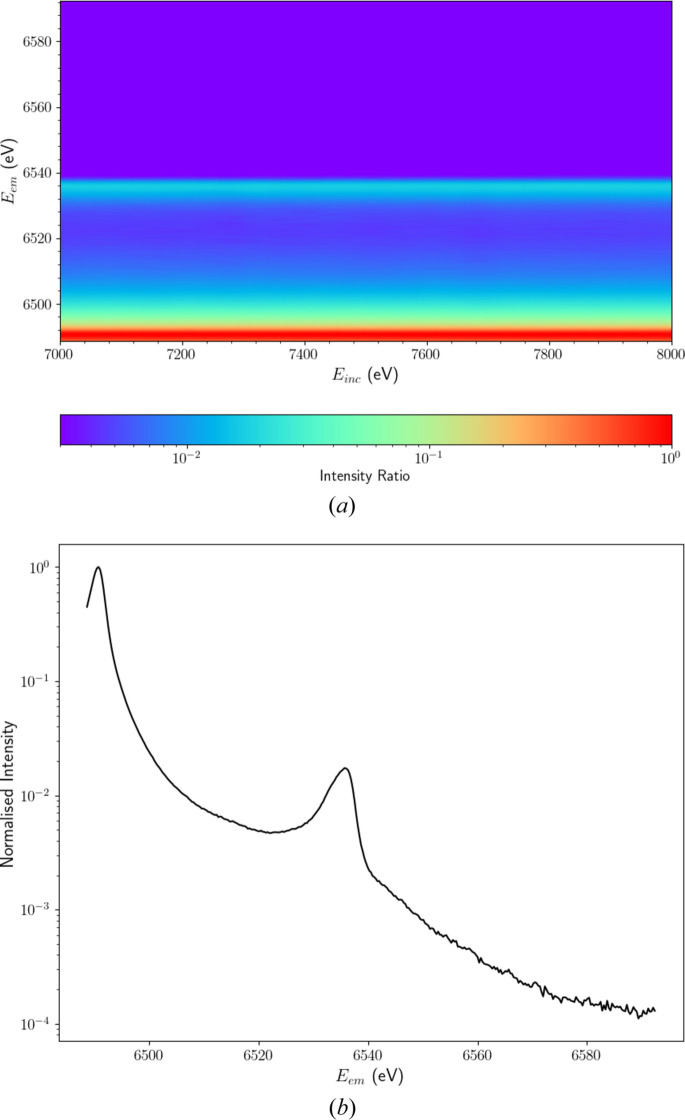
(*a*) XR-HERFD contour plot of the average ratio of fluorescent counts to upstream ion-chamber counts of the crystal analysers in Mn metal. Two spectral features are observed. The first is the *K*β_1,3_ transition at an emission energy (*E*_em_) of 6490.4 eV, which arises due to a 3*p* electron filling the vacancy of the core photoelectron. The second feature is the *K*β_2,5_ transition centred at *E*_em_ = 6535 eV, which arises predominantly due to 3*d* electrons filling the core vacancy. The incident energy (*E*_inc_) ranges are well above the *K* edge and hence the relative intensity of the two processes observed remains consistent. The structure of these two spectral features is evident in more detail in panel (*b*), which portrays an aggregated HERFD-XES slice of the contour plot from *E*_inc_ = 7000 to 7160 eV. A logarithmic scale is required on the vertical axis to depict the *K*β_2,5_ transition in detail, which is suppressed by several orders of magnitude relative to *K*β_1,3_ due to its primary origin from quadrupole transitions. Notably, there are no spectral features evident in the high-energy (*E*_em_ > 6540 eV) region.

Fig. 2 ▸(*a*) displays the fully processed XR-HERFD intensity map. Two characteristic peaks are evident and presented in further detail in Fig. 2 ▸(*b*). The first peak at *E*_em_ = 6490.4 eV corresponds to the *K*β_1,3_ transition, arising from electric dipole transitions when a 3*p* electron fills the 1*s* core hole. The second peak, at *E*_em_ = 6535 eV, represents the *K*β_2,5_ transition, primarily composed of quadrupole transitions from the 3*d* to 1*s* orbitals. Due to electronic selection rules, this peak is suppressed by several orders of magnitude relative to *K*β_1,3_.

**Figure 3:**
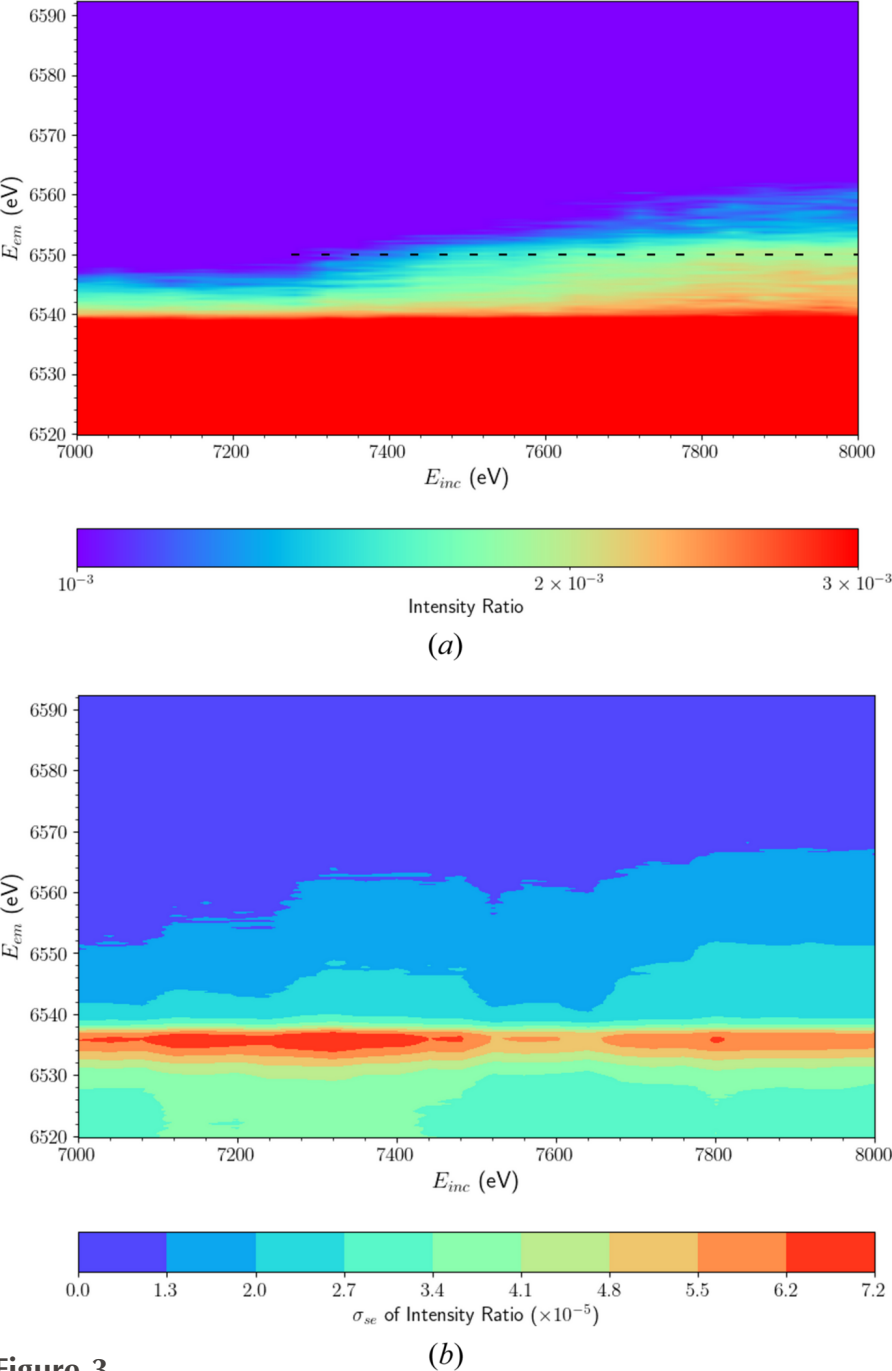
(*a*) XR-HERFD contour plot, analogous to Fig. 2, with restricted domain to focus on the high-energy region. The emergence of a spectral feature is evident past an incident energy *E*_inc_ = 7280 eV, marked by the dashed line at an emission energy *E*_em_ = 6550 eV. We attribute this spectral feature to a *K*β satellite of Mn. (*b*) Propagated standard error (σ_se_) for the XR-HERFD map of intensity ratios in panel (*a*).

In line with our objective to resolve novel satellites in manganese *K*β, we examine the high *E*_em_ region of the XR-HERFD map in more detail in Fig. 3 ▸(*a*). A faint structure emerges past *E*_inc_ ≃ 7300 eV in the region between *E*_em_ = 6540 and 6560 eV. This previously unobserved physical phenomenon in manganese *K*β highlights the effectiveness of XR-HERFD in resolving processes well above the absorption edge. Due to the separation of the analyser crystal images on our detector apparatus, we can display standard error uncertainties (σ_se_) as in Fig. 3 ▸(*b*), quantifying the precision of our results.

## 4. Empirical isolation of the satellite

Whilst Fig. 3 ▸(*a*) appears to reveal a new satellite in manganese *K*β, garnering any insights on its structure or characteristics appears at this stage to be incredibly difficult and imprecise. Shake-off processes are generally weak compared with their single-body counterparts, although the meaning of this remains quite unclear in the experimental literature and theoretical investigations are only now beginning to make verifiable estimates. Here, the isolation of the satellite is exacerbated due to the intrinsically lower intensity of *K*β compared with *K*α and the anticipated dominance of Auger decay in the shake-off channel (Dean *et al.*, 2024 ▸).

We address this issue using data processing with a background subtraction. Here, the reference background is defined as processes which are stable with respect to increasing *E*_inc_ at some reference energy well above the *K* edge. To define this background spectrum, we first posit a lower bound for the onset of the satellite process. Based on observations of Fig. 3 ▸(*a*) this occurs prior to *E*_inc_ = 7280 eV, and so we define our background as an aggregate of the normalized HERFD-XES slices from *E*_inc_ = 7000 to 7160 eV. This corresponds to the emission spectrum seen in Fig. 2 ▸(*b*), incorporating the *K*β_1,3_ and *K*β_2,5_ transitions and their high-energy tails. Note we do not assume that this is stable, only that it is relatively and sufficiently stable.

**Figure 4:**
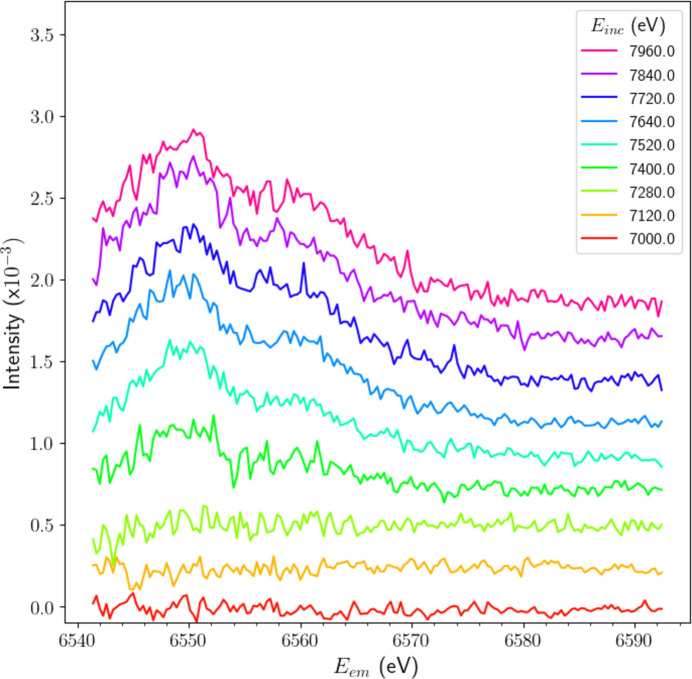
Stack plot of a collection of HERFD-XES slices following the subtraction of the *K*β background. The background, which corresponds to the stable transitions in this incident energy (*E*_inc_) range, is evident in Fig. 2(*b*). The signal-to-noise ratio is improved due to the elimination of electronic noise in the data (Appendix *A*[App appa]), selective use of the eight highest-resolution analyser crystals (Appendix *B*[App appb]) and the naturally suppressed intensity of the *K*β satellite. While only noise fluctuations are evident at low *E*_inc_, two distinct and prominent peaks emerge and remain evident in the substructure beyond 7280 eV. This is definitive evidence for the evolution of the satellite.

By subtracting this (scaled) background spectrum from the normalized XR-HERFD data, we are left with a clear depiction of spectral features that emerge only well above the *K* edge and reference incident energies *E*_inc_. Fig. 4 ▸ presents a collection of the resulting HERFD-XES slices after subtraction. The signal-to-noise ratio is improved significantly and we can clearly see the emergence of a consistently shaped dual-peak substructure past *E*_inc_ = 7280 eV. The level of noise in Fig. 4 ▸ still appears large, with quite significant statistical uncertainty on a given pixel point in the map, but we note that the structure appears to be both consistent and becoming stronger with increasing energy *E*_inc_. The apparent improvement is a consequence of the elimination of electronic noise in the XR-HERFD data (Appendix *A*[App appa]), selective use of the eight highest-resolution analyser crystals (Appendix *B*[App appb]) and combining statistics. That we are able to resolve the satellite’s structure and evolution, given these circumstances and the relatively low intensity of the satellite, is a triumph for XR-HERFD.

**Figure 5:**
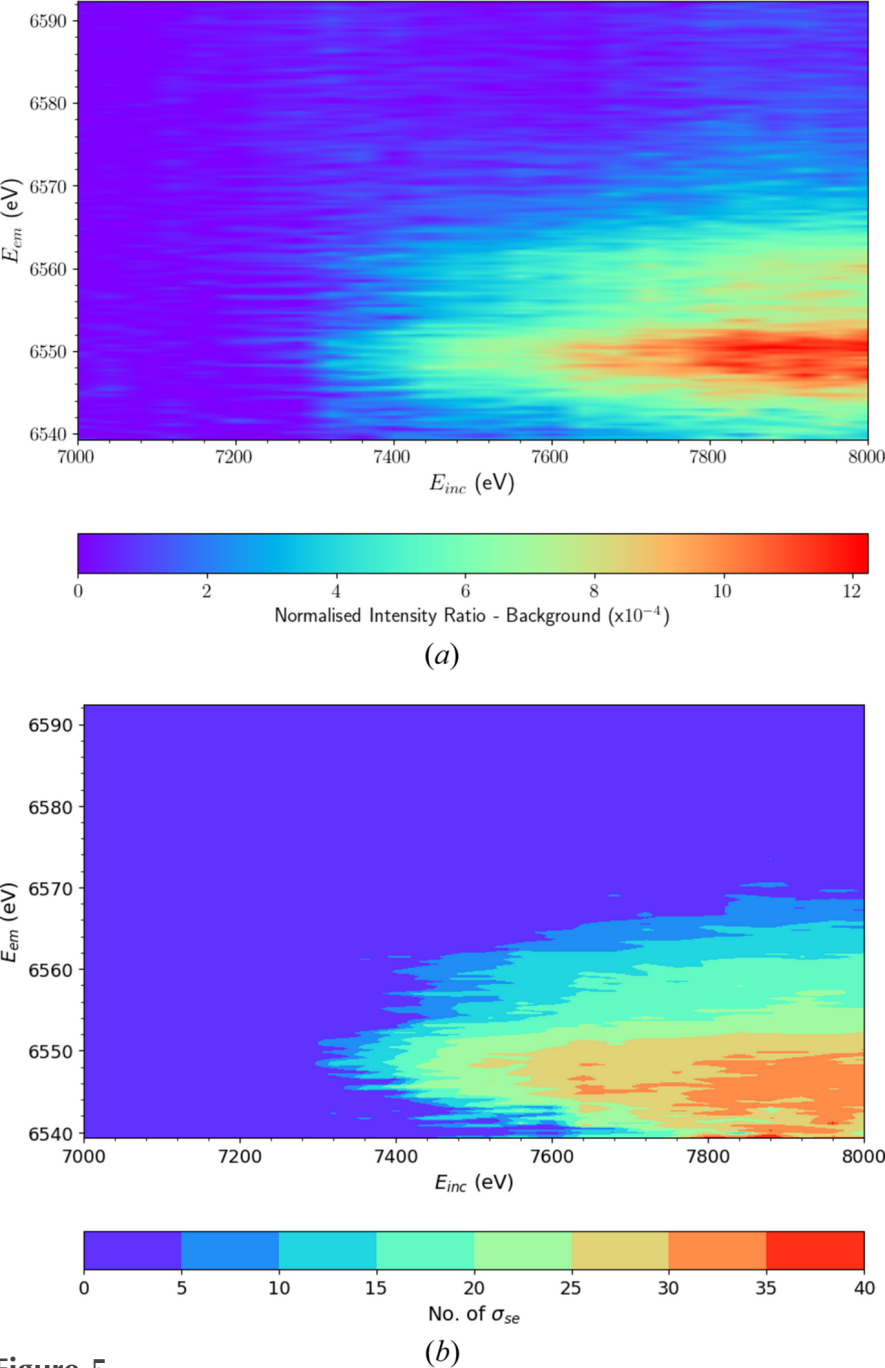
(*a*) XR-HERFD map beyond *K*β_2,5_ after background subtraction using eight analyser crystals. A dual-peak substructure clearly emerges beyond an incident energy *E*_inc_ = 7280 eV and is distinct from the noise fluctuations seen at higher emission energies (*E*_em_). (*b*) XR-HERFD contour plot of the significance of the satellite region, displaying the number of standard errors (σ_se_) following subtraction of the background. The number of standard errors per pixel around the peak *E*_em_ of the satellite, ∼6550 eV, increases from effectively 0 prior to the onset to over 30 at the maximum *E*_inc_ for just a single pixel. This is well beyond the usual threshold for discovery (3–6 σ_se_ for the total integrated signal) and corresponds to a total signature of 652 σ_se_ in the satellite region. This is a new satellite.

The background-subtracted XR-HERFD map is depicted in Fig. 5 ▸(*a*). Here, the satellite is clearly significant and distinct from residual noise and more clearly identified.

The number of standard errors for each pixel in Fig. 5 ▸(*a*) is depicted in Fig. 5 ▸(*b*). The significance increases as the satellite evolves and is above 20 at each pixel along the satellite peak. This lies far above the typical level for discovery (σ_se_ = 3 to 6) and results in a total integrated significance of 652 σ_se_.

**Figure 6:**
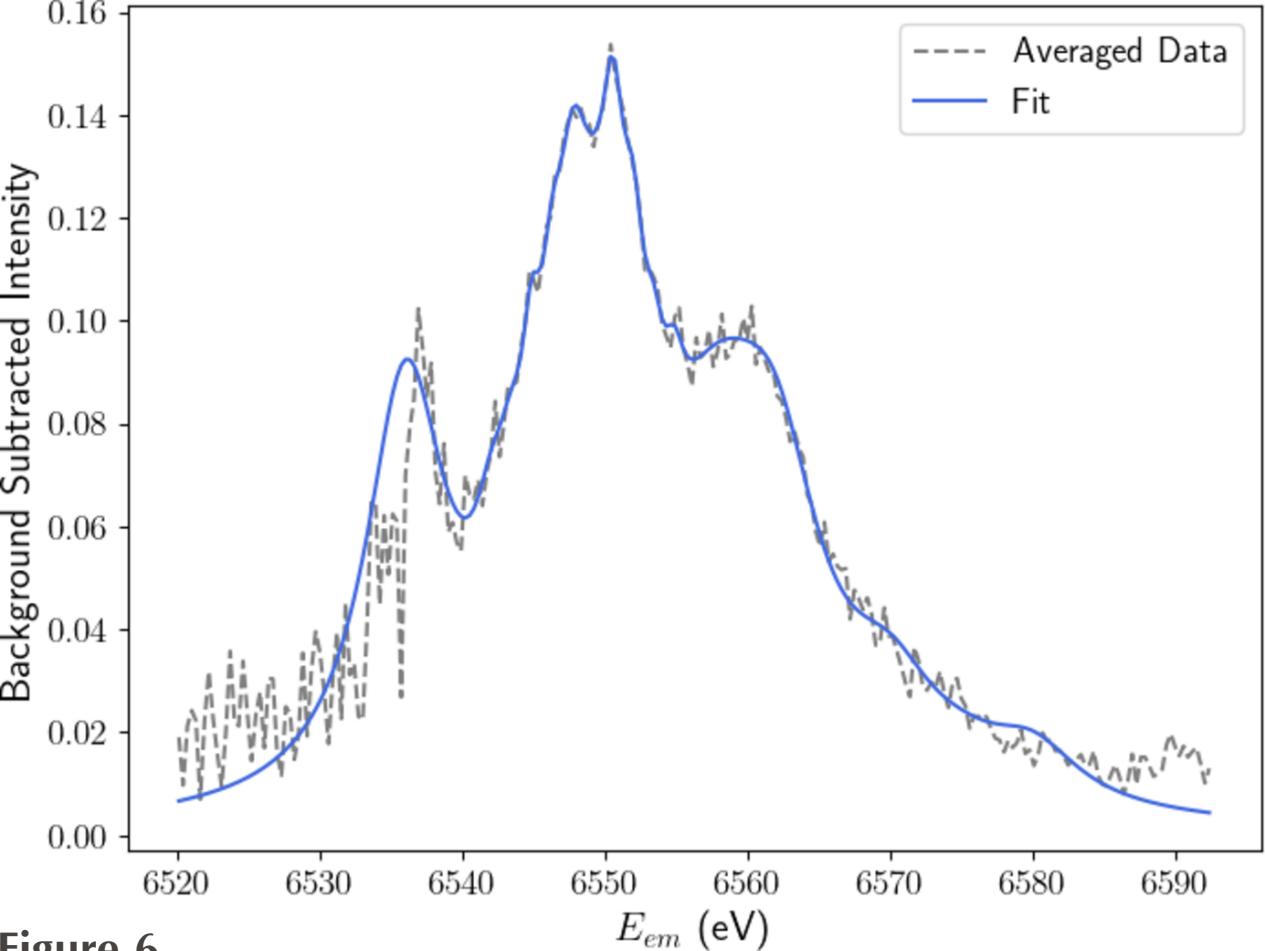
Extracted satellite, obtained by averaging across the background-subtracted spectra for incident energies from 7840 to 8000 eV. This is then fitted empirically with a sum of Lorentzians, concentrated around prominent features in the data, to obtain a detailed initial guess for the structure of the satellite. Notable features include a main double peak and prominent low- and high-energy shoulders. Note that this extraction does not use the full data set but uses the strongest statistical representation of the new spectrum. Empirical extraction is prone to the propagation of systematic errors or noise which may distort the structure of novel spectral features. We thus turn to statistically rigorous methodologies in Section 5 to extract detailed structural insights.

We need to do better, so we average the background-subtracted spectrum from *E*_inc_ = 7840 to 8000 eV to yield a better defined representation of the satellite structure. Across this higher energy range, the satellite intensities begin to stabilize. This representation is evident in Fig. 6 ▸. For visual clarity and to suppress noise, we fitted this structure with a sum of Lorentzians weighted around prominent features in the spectrum.

The satellite appears to consist of a main peak with both low- and high-energy shoulders, while the main peak itself exhibits an intriguing double apex. This detailed fine structure suggests that the satellite has origins in multiple physical phenomena, as has been concluded for the *K*α satellite (Sier *et al.*, 2024 ▸). Note that this does not use the full data set but uses the strongest statistical representation of the new spectrum. It might be recognized that the background subtraction technique is prone to propagating systematic errors or noise into the XR-HERFD data, particularly given the low intensities investigated here, which could mislead researchers or conceal key structural insights. Hence, we turn to a more statistically rigorous and complete approach.

## 5. Investigation with principal component analysis

To extract detailed structural information on the satellite, we employ the machine-learning processing technique known as principal component analysis (PCA). By transforming the original data set into a new set of uncorrelated variables (the ‘principal components’, PCs) through linear combinations that maximize variance of the next component, PCA enables dimensionality reduction without significant information loss, making complex data sets easier to analyse and interpret (Jolliffe, 2002 ▸; Ramsay & Silverman, 2006 ▸; Witten *et al.*, 2016 ▸; Jolliffe & Cadima, 2016 ▸; Hao & Ho, 2019 ▸). To perform PCA we use the *scikit-learn* (*sklearn*) library in Python (Pedregosa *et al.*, 2011 ▸; Buitinck *et al.*, 2013 ▸) with some minor modifications. A complete description of our PCA procedure is presented in Appendix *C*[App appc].

**Figure 7:**
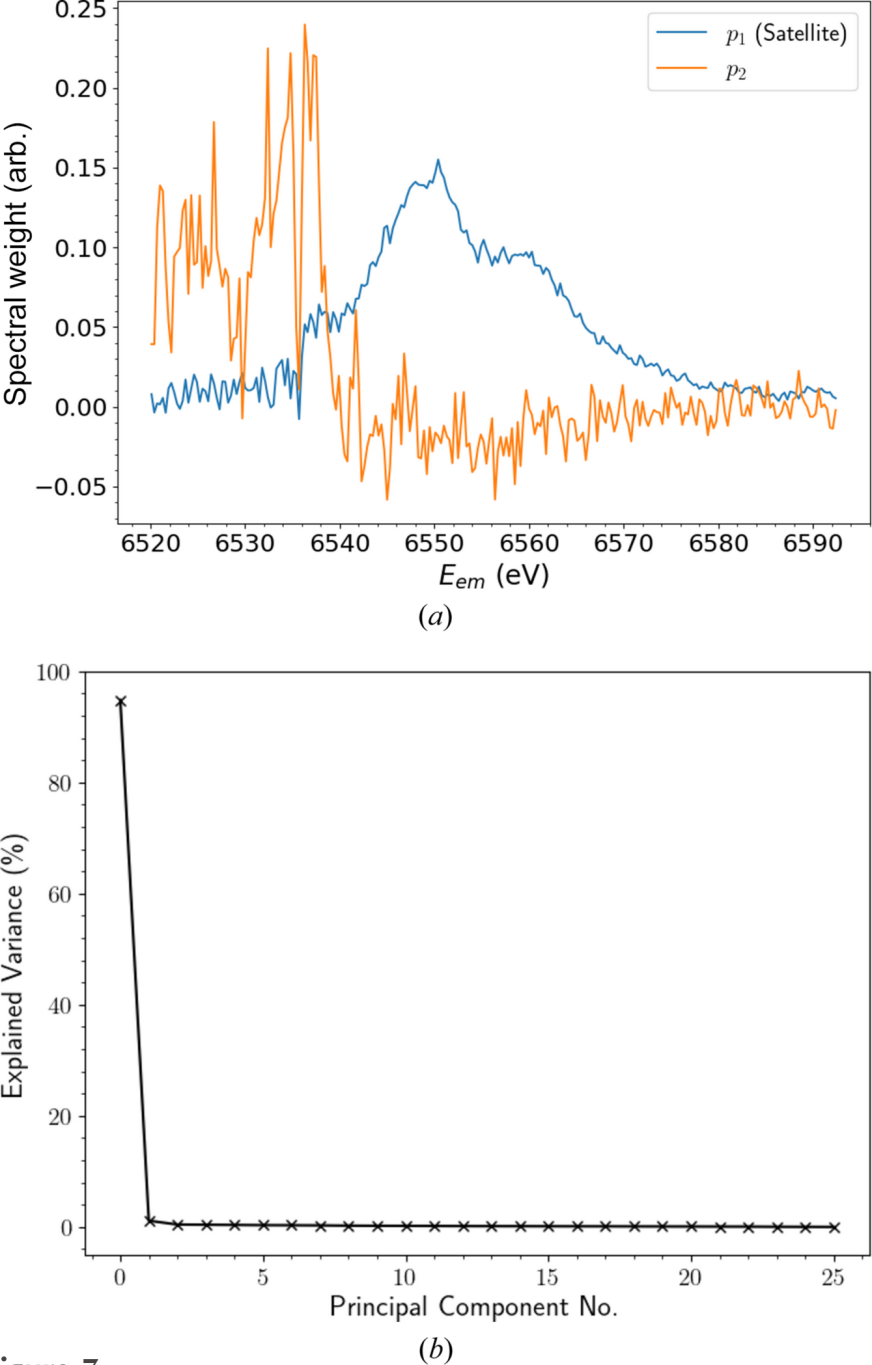
(*a*) Weights of the first two principal components (PCs) as a function of emission energy *E*_em_. A visual inspection makes it immediately apparent that the first PC can be interpreted physically as the satellite (*p*_1_) and the second (and higher) PCs correspond to (correlated) noise (*p*_2_). The satellite closely resembles the initial estimate in Fig. 6 but with increased structural detail. The first component of noise has a higher magnitude in the vicinity of the *K*β_2,5_ peak, a consequence of the larger intensities and hence noise in that region. (*b*) Fraction of the total variance explained by each subsequent PC. The satellite component accounts for over 95% of the variance across the background-subtracted XR-HERFD data set. Hence we can focus on the satellite component without sacrificing any loss of information.

Fig. 7 ▸(*a*) depicts the weight (loading) of each *E*_em_ in the first two PCs. This reveals clear physical interpretations for both. Those of the first PC (*p*_1_) correspond to the satellite, closely resembling the structure extracted in Fig. 6 ▸. We shall thus refer to *p*_1_ as the satellite (component). The second PC (*p*_2_) and above possess similar structures, dominated by correlated residual noise in the *K*β tail, as indicated by the higher intensity around *E*_em_ = 6535 eV, where the peak of *K*β_2,5_ resides. When extended to include the entire range of *E*_em_, each of these higher-order components reveals dominant features at ∼6490 eV, corresponding to the *K*β_1,3_ peak. Given that their only significant features emerge within characteristic spectral profiles, we can attribute *p*_2_ and above to correlated noise. Additional physical features may be hiding within these components, but show no prominent contributions to satellite spectra.

The impact of pre-processing artefacts is evident in the variation of component weights based on the selected range of *E*_em_. The choice of normalization can substantially affect the apparent variance within characteristic spectral features, which contribute less to the overall variance given their stability in the high *E*_inc_ region of our data set. Notably, correction for Bragg angle variation through binary data splicing and removal of intensity aberrations (Appendices *A*[App appa] and *B*[App appb]) have enhanced the physical interpretability of our components, which may otherwise have been dominated by experimental systematics rather than physical spectral variation.

Fig. 7 ▸(*b*) reveals the proportion of variance across the background-subtracted data explained by each PC. Clearly, the satellite is the most prominent component, explaining >95% of the total variance, while each of the noise components contributes 1% or less. In the remainder of our analysis, we can thus neglect or reject *p*_2_ and above and focus solely on the satellite component without significant information loss.

**Figure 8:**
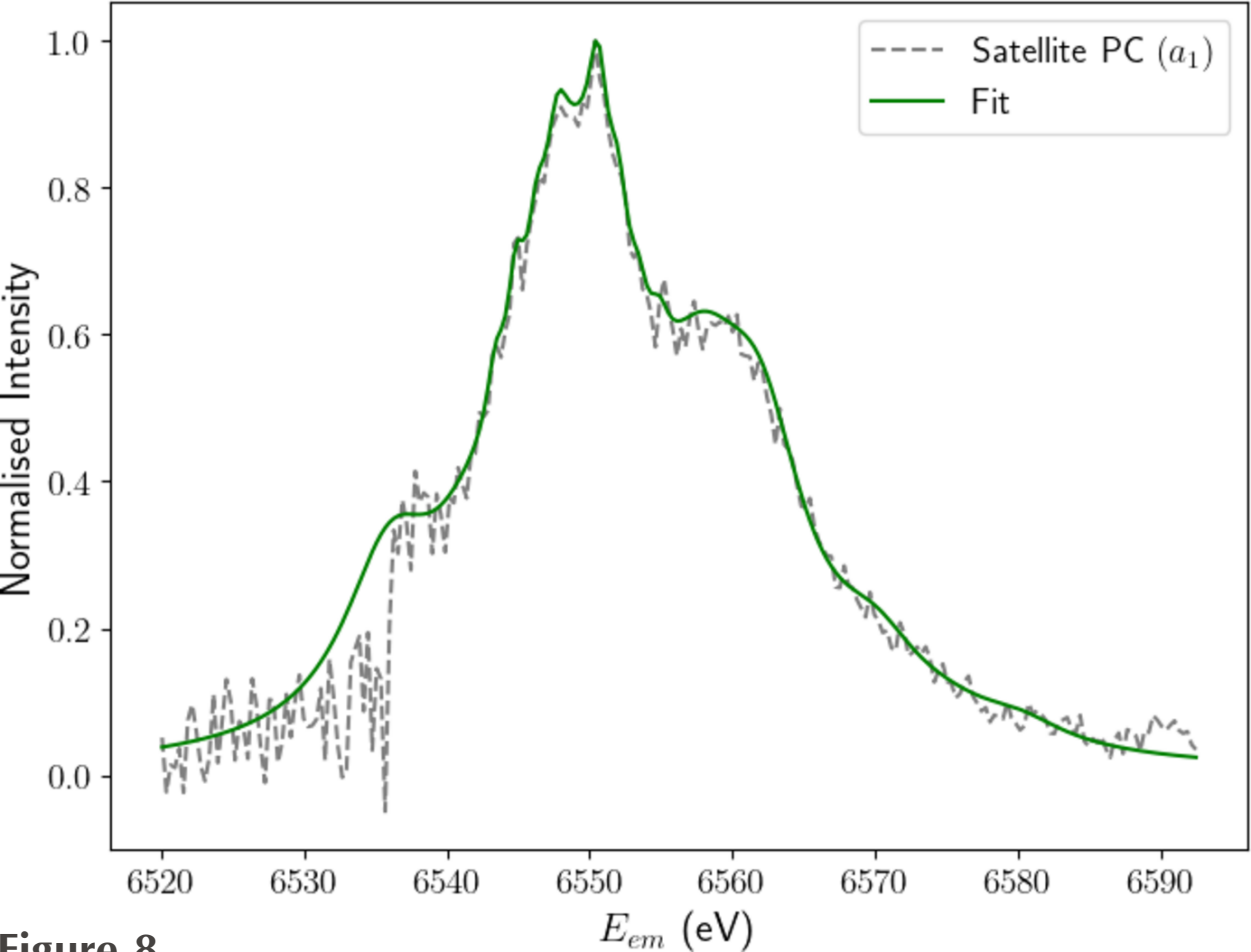
The satellite principal component (*p*_1_) fitted with a sum of Lorentzians to emphasize key features. The structure appears similar to that derived in Section 4, but with greater detail, revealing a main dual peak with shoulders on the high- and low-energy sides. The level of fine structure depicted suggests that numerous components contribute to the overall satellite structure, which is indicative of a shake process.

We garner further structural insights from the satellite component by fitting a sum of Lorentzians (Fig. 8 ▸). Note that in Fig. 6 ▸ we were able to extract a detailed representation of the most important *p*_1_ component using our ‘manual’ approach, yet also note that there are limitations of interpretation with any such approach including PCA. The resulting structure closely resembles our fit of the averaged empirical satellite in Fig. 6 ▸ but with significantly less noise, in part because the whole data set can be used. This demonstrates the effectiveness of applying PCA to XR-HERFD data sets, as it allows us to extract key evolutionary components and remove almost all of the (randomly) distributed residual noise, allowing for physical interpretation of the resulting structure.

As discussed in Section 4, the satellite component possesses significant fine structure. A prominent double peak stands at *E*_em_ ≃ 6550 eV, straddled by high- and low-energy shoulders at 6560 and 6535 eV, respectively. The low-energy shoulder was not consistently resolved in the background-subtracted data in Figs. 4 ▸, 5 ▸(*a*) and 6 ▸ due to noise fluctuations in the *K*β_2,5_ tails. Even in the satellite PC, the shoulder remains somewhat ambiguous. The dominance of correlated noise in each of the *p*_2_ and above components in the region of the low-energy shoulder is a likely cause for this discrepancy. Intensities are two orders of magnitude greater in the *K*β_2,5_ profile than in the satellite and hence any fluctuations in this region are bound to obscure the resolution of evolving satellite features. Regardless, the entirety of the structure is resolved in much greater detail than in our earlier analysis, due to the combination of statistics.

The level of fine structure evident in the satellite component suggests it has multiple near-degenerate physical origins, typical of a shake-off satellite. Notable features offer insights into the origin of the process. The satellite is displaced by ∼60 eV in *E*_em_ from the *K*β_1,3_ peak and the onset is ∼700 eV past the *K* edge. In addition to the low intensity of the process, these factors indicate that the physical origin of the satellite is *n* = 2 shake-off events in the *K*β spectrum. Specifically, we expect either a 2*p* or 2*s* electron to be ‘shaken-off’ during the photoionization of a core electron, before a 3*p* electron fills the core hole in a high-energy environment, resulting in the emission of a photon non-degenerate to the characteristic *K*β_1,3_ spectrum. This aligns with theoretical predictions for manganese (Dean *et al.*, 2024 ▸).

**Figure 9:**
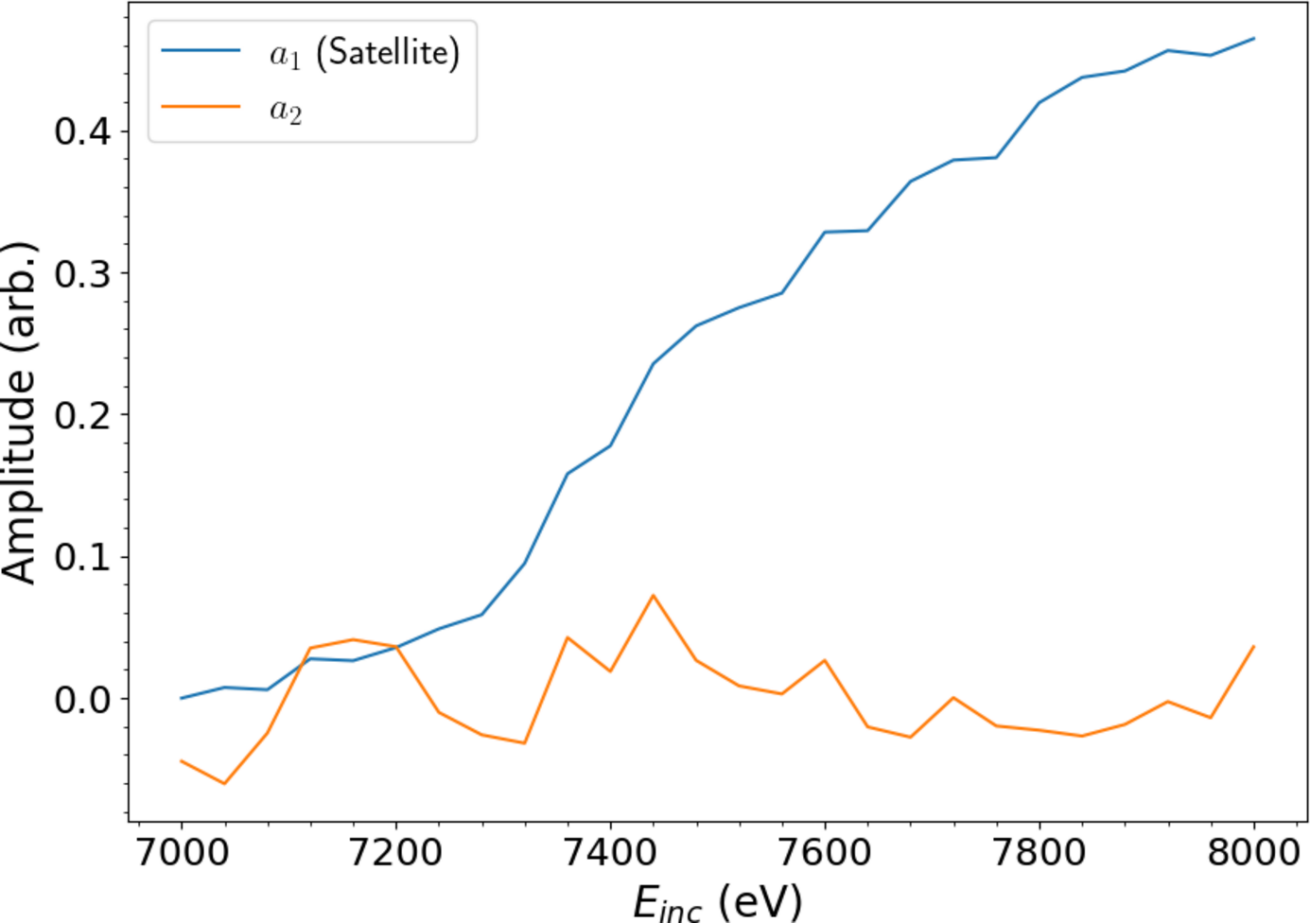
Amplitudes of the first two principal components of the background centred data. These correspond to linear combinations of the background-subtracted XR-HERFD data weighted by the satellite and noise components defined in Fig. 7. The monotonic increase of the satellite amplitude past an onset around an incident energy *E*_inc_ = 7200 eV is indicative of many-body process evolution. This begins to slow past 7800 eV, as was observed with an analysis of empirical data in Section 4. The amplitude of the noise component oscillates without any clear pattern around 0, confirming that it does not correspond to any significant or persistent structure.

The weights in Fig. 7 ▸(*a*) are applied to the background-subtracted data to produce linear combinations which are representative of the amplitude of a given PC at each *E*_inc_. Depicted in Fig. 9 ▸, the amplitude of the satellite component (*a*_1_) increases monotonically from 0 to a maximum as *E*_inc_ of the HERFD-XES slices passes 7200 eV, indicating that the satellite feature *p*_1_ is growing stronger, evolving with increasing *E*_inc_. This is equivalent to the growth of a consistent process observed in Fig. 4 ▸, but with a reduction in noise and dimensionality. The significance of this observation is highlighted by the amplitudes of the *a*_2_ component and above, which oscillate around zero without any discernible pattern. This confirms that these components arise from correlated noise contributions and not from physical phenomena that contribute prominent variance to the *K*β background spectra. An application of *k*-means clustering to the data following PCA projection reveals much the same insights, but separates the HEFD-XES slices rather arbitrarily with regard to *a*_1_ and *a*_2_ amplitudes, limiting physical interpretability.

## 6. Evolution of the satellite

The form of the amplitudes in Fig. 9 ▸ clearly indicates the evolving intensity of the satellite past an onset at approximately 7200 eV. Near the onset, *E*_inc_ is approximately equal to the combined binding energies of the two ejected electrons, and hence the probability of ejection for the secondary photoelectron is low. Since the primary photoelectron exits the atomic system with low kinetic energy, the timescale for its ejection from the atom is large and thus the mechanisms behind the shake-off event must be considered through Coulomb interactions, necessitating solutions to the time-dependent Schrödinger equation in an incredibly complex atomic system (Mukoyama *et al.*, 2009 ▸; Valenza *et al.*, 2017 ▸).

As the incident photon energy increases, the timescale of the primary photoelectron ejection continues to decrease as a result of its greater kinetic energy and velocity, resulting in a gradual increase in shake-off probability, as was observed experimentally through the increase in fluorescence intensity. Well past the satellite onset, the kinetic energy of the primary photoelectron becomes so large that its ejection can be treated as instantaneous and without interaction with the remaining atomic system in what is known as the sudden approximation (Dean *et al.*, 2024 ▸). In the sudden approximation, the mechanism of the shake process can be derived entirely through the relaxation of atomic orbitals following ionization of the primary electron (Kochur & Popov, 2006 ▸).

Under the sudden approximation, the shake-off probability approaches a maximum value, referred to as *P*(∞). This limiting shake probability could be interpreted as a basis for the current representation of 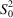 (Roy *et al.*, 2001 ▸). Contemporary XAFS models and analysis consider a constant probability of shake events, which is fundamentally in disagreement with experimental results observed here and elsewhere (Tran *et al.*, 2023 ▸; Sier *et al.*, 2024 ▸). To demonstrate this, we analyse and model the evolution of the satellite.

### 6.1. Extracting a functional form for many-body evolution

By applying PCA to our XR-HERFD data set, we have obtained a representation for the structure of the satellite significantly bereft of correlated noise [Fig. 7 ▸(*a*)] and an initial guess for the functional form of the satellite evolution (Fig. 9 ▸). To test the robustness of the PC satellite structure, we implement a form of principal component regression, which attempts to express a set of data as a weighted linear combination of its PCs (Witten *et al.*, 2016 ▸). As Fig. 7 ▸(*b*) depicts, the satellite (*p*_1_) explains >95% of the variance from the refined *K*β background profile (μ′). Hence we can ignore contributions from the correlated noise components and attempt to recreate the XR-HERFD data (*X*) using *p*_1_ and our refined *K*β background profile μ′ (defined in Appendix *C*[App appc]),
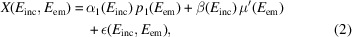
where ε is a residual term. To test the effectiveness of this representation (which we term the PCA model), we compare the performance of an alternative model. This model replaces the relevant terms in equation (2) with the forms of the background [Fig. 2 ▸(*b*)] and satellite (Fig. 6 ▸) we determined in Section 4 by aggregating relevant segments of the HERFD-XES slices in *X*. We denote this model as the aggregate model.

**Figure 10:**
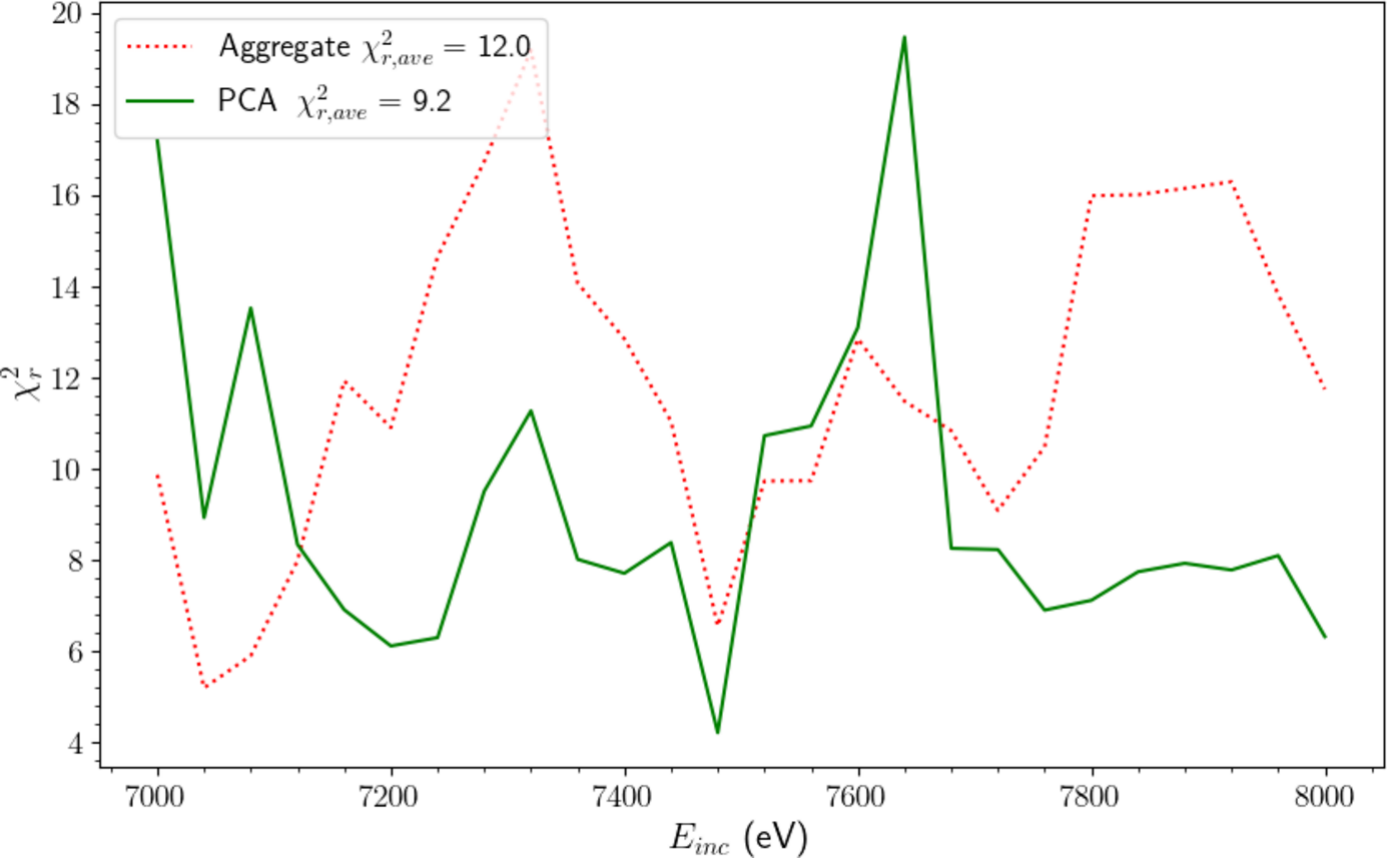
Performance of two separate models fitted to HERFD-XES slices in the XR-HERFD data evaluated across the entire range of incident energies *E*_inc_. The PCA model follows the functional form in equation (2), employing the satellite (*p*_1_) and background (μ′) extracted using PCA in Section 5. The aggregate model substitutes the relevant terms in equation (2) with the satellite (Fig. 6) and background [Fig. 2(*b*)] we obtained by averaging over relevant HERFD-XES slices in Section 4. The PCA model frequently outperforms the aggregate model, especially past *E*_inc_ ≃ 7200 eV, yielding an average 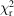 25% lower than the aggregate model. This is a strong indicator that the PCA algorithm accurately captures the satellite structure.

Fig. 10 ▸ presents the results of this comparison. While the aggregate model provides a better fit at low *E*_inc_, the PCA model frequently outperforms it as *E*_inc_ progresses beyond the satellite onset (∼7200 eV). This is demonstrated by the PCA model yielding an average goodness-of-fit 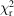 of 9.2, which is approximately 25% lower than that of the aggregate model (12.0). Hence the parameters extracted using PCA yield an improved and accurate representation of the XR-HERFD data. Therefore, we adopt α_1_ from equation (2) as the functional form to describe the evolution of the satellite intensity.

### 6.2. Comparison of evolutionary models

We investigate the evolution of the satellite (component) from our observed data compared with theoretical predictions from three literature models for satellite or quantum mechanical process evolution. The first, the most commonly cited quantum evolution model (Thomas, 1984 ▸), is
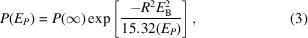
where *P*(∞), defined here as the change in potential due to the ejected core electron divided by the binding energy squared, is the limiting shake probability in the sudden approximation, *R* is the atomic size corresponding to the radius of the shake-off orbital in ångströms, *E*_B_ is the binding energy of the shake electron, *E*_*P*_ = *E*_inc_ − *E*_edge_ − *E*_B_ is the energy of the shake electron and the constant is given by 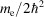 in units of electronvolts and ångströms.

Roy *et al.* (2001 ▸) developed an alternative form for quantum evolution, employing the Slater form of one-electron wavefunctions and assuming the time dependence carries an exponential form,
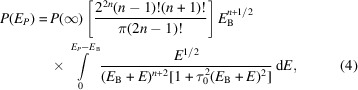
where *n* is the principal quantum number of the shake shell, 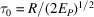 is the characteristic time for the interaction and *P*(∞) is obtained by performing the high-energy limit of the integral and setting τ_0_ = 0. All variables in the Roy formalism are expressed in Hartree atomic units (*m*_e_, ℏ, *e*, 4πε_0_ = 1), which we convert to electronvolts and ångströms when presenting our results.

Mukoyama *et al.* (2009 ▸) adapted the Thomas model using the exponential time dependence from Roy to obtain 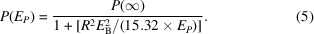


While Mukoyama and Thomas considered bound–bound transitions (shake-up) and Roy bound–free (shake-off), each model is sufficient to describe the evolution of shake probabilities. We examine the evolution of *I*_sat_/*I*_*K*β_ as a function of *E*_inc_, which reflects the probability of an *n* = 2 satellite transition relative to the entire *K*β spectrum. We determine this by extracting α_1_ from equation (2) and multiplying it by the satellite area as a fraction of the total *K*β spectrum 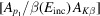. 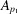 is the area of the satellite PC defined in Fig. 8 ▸, *A*_*K*β_ is the area of the PCA-extracted background μ′ extended to incorporate the full *K*β spectrum and β(*E*_inc_) is the scaling parameter of the background from equation (2).

**Figure 11:**
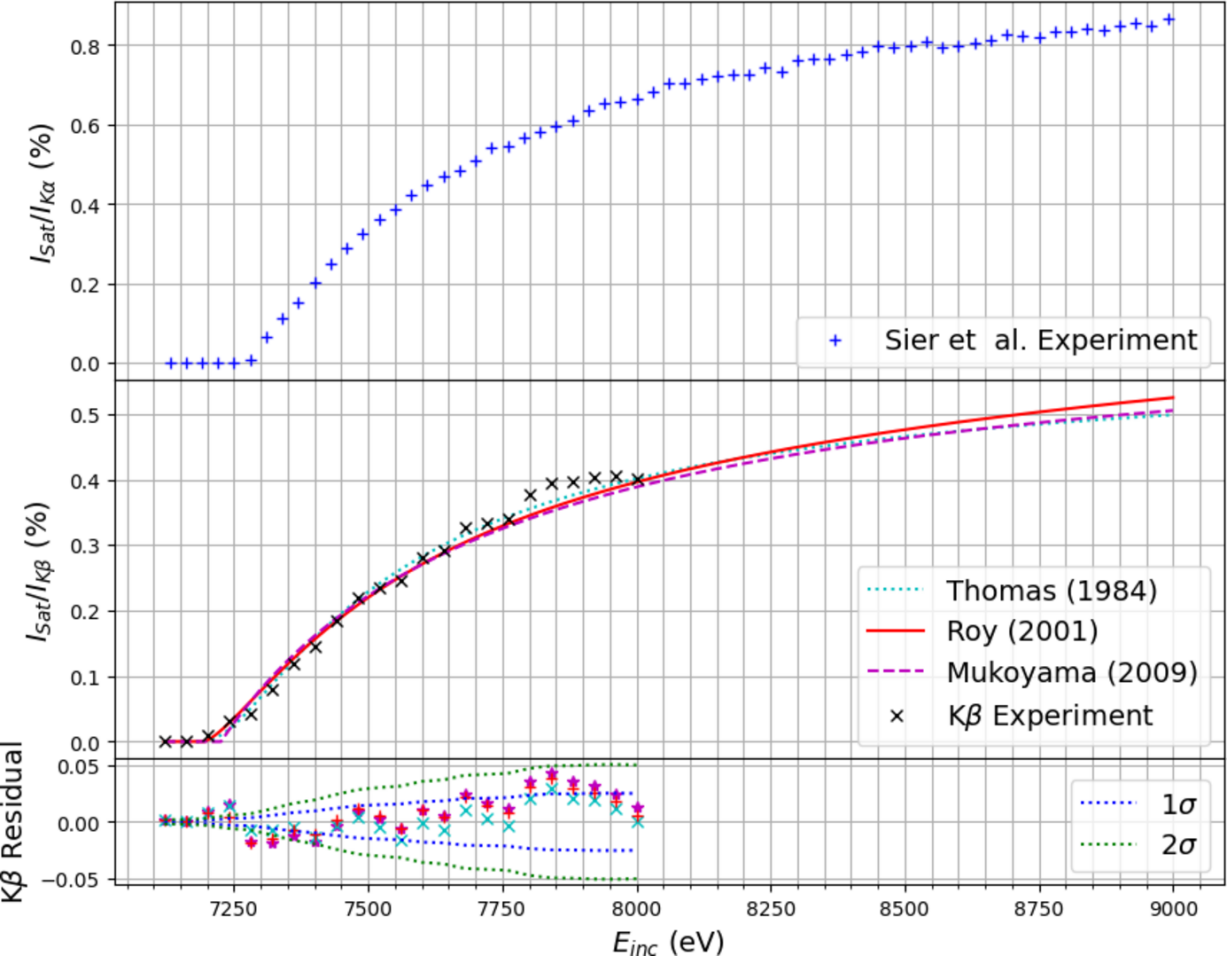
(Top) Evolution of the *K*α satellite intensities from Sier *et al.* (2024 ▸). (Middle) Evolution of the relative intensity of the *K*β satellite extracted from experimental data after PCA processing. The line shape of the *K*β evolutionary curve is remarkably similar to that of the *K*α satellite. Note, however, the relative intensities are consistently a factor of 1.5–2 times lower and the onset appears to be 50–100 eV earlier. The results for *K*β are fitted to three models describing the evolution of quantum probability using the parameters in equation (2). (Bottom) Residuals of the *K*β satellite. The Thomas model consistently returns the lowest residual to the experimental data. However, evolution of the experimental intensity ratios begins to slow past an incident energy *E*_inc_ = 7800 eV, which disagrees with the models.

We fitted the models to our evolution data while leaving *R*, *P*(∞) and *E*_B_ as free parameters and extracted statistical uncertainties for each. The results are depicted in Fig. 11 ▸, alongside data for the *K*α satellite from Sier *et al.* (2024 ▸). Clearly, many-body transitions do not display a sharp jump in intensity after their onset, deviating significantly from the well documented step-function-like increase claimed in the near-edge region of single-body spectra. Instead, they gradually evolve after the onset incident energy has been reached, slowly increasing in intensity over a spread of incident energies before approaching a maximum intensity.

The performance of each model is quantified by the fitting parameters (Table 1 ▸). Reference values were calculated using the multi-configuration Dirac–Hartree–Fock (MCDHF) method with a relativistic quantum electrodynamics approach in the *General Relativistic Atomic Software Package* (*GRASP*) by Sier *et al.* (2024 ▸). While these were originally derived for analysis of the *K*α satellite, the parameters *E*_B_, *P*(∞) and *R* describe an *n* = 2 shake-off event independent of the subsequent decay channel. Hence, they serve as suitable comparative estimates for the *K*β satellite. The quantitative performance of the three models can be compared with their results for the *K*α satellite.

Δ*E* is the difference between the fitted binding energy (*E*_B_) of the *n* = 2 shake orbital and that computed using *GRASP*. *GRASP* yields estimates of 706.53 eV and 721.05 eV for the 2*p*_3/2_ and 2*p*_1/2_ orbitals, respectively.

Resolving the onsets of individual satellite components with statistical robustness is challenging due to low signal-to-noise ratios and near-degeneracy. Consequently, we analyse the evolution of the satellite structure as a whole based on the lowest binding energy component (2*p*_3/2_). Several estimates for the *K* edge exist in the literature. Computationally, *GRASP* returns a value of 6547.1 eV and Huang *et al.* (1976 ▸) predict 6549.8 eV. The most up-to-date experimentally measured value (at 77 K) obtained by Kraft *et al.* (1996 ▸) and Chantler *et al.* (2025 ▸) is 6537.67 eV, which we take as the 1*s* binding energy in our calculations for the onset. Intriguingly, the theoretical predictions are ∼10 eV higher than accurate experimental measurements, which may be a result of neglecting pre-edge structure.

Sier *et al.* (2024 ▸) predicted an onset at 7244.2 eV for the 2*p* shake-off, while the Thomas, Roy and Mukoyama models return 7139.3 eV, 7190.7 eV and 7224.2 eV, respectively, using fitted values for the binding energy in *K*β, all noticeably lower than *GRASP*.

While experimentally defining a precise onset is difficult, Figs. 9 ▸ and 11 ▸ suggest a range of 7200–7280 eV. The onset for *K*α appears to be ∼50–100 eV higher. This may be a consequence of the lower signal-to-noise ratio in *K*β contributing to intensities in the satellite region. Both the Roy and Mukoyama models are relatively consistent with experimental observations, but the Thomas model significantly underestimates the onset, falling below even a lower bound estimate derived from the unperturbed binding energy of the 2*p*_3/2_ orbital (638.7 eV; Thompson *et al.*, 2001 ▸). This discrepancy highlights the limitations of the Thomas model in predicting the form of satellite evolution near the onset.

*GRASP* returned a value of 0.098 Å for the expectation value of the 2*p* shake orbital effective radius (*R*). Among the models considered, the Thomas model (0.105 Å) exhibits the closest agreement, as was also observed for the *K*α satellite. The Roy (0.129 Å) and Mukoyama (0.131 Å) models return significantly larger values.

*GRASP* yielded a limiting shake-off probability *P*(∞) of 1.108%. Fig. 11 ▸ suggests a lower experimental value for the limit of *I*_sat_/*I*_*K*β_, with evolution slowing around 0.4% beyond *E*_inc_ = 7800 eV. The three models predict *P*(∞) within the range of 0.6%–0.8%, contrasting with the *K*α satellite where all models were within 5% of the *GRASP* estimate. The most significant contribution to this discrepancy is the narrow range of *E*_inc_ recorded here (7000–8000 eV) which extends only ∼700 eV beyond the onset, as opposed to thousands of electronvolts in data for *K*α (7000–10000 eV). The models are thus unreliable in predicting the evolutionary behaviour of the satellite well beyond the onset into the sudden limit, as shown by the relatively large statistical uncertainty (10%–20%) on *P*(∞) in each of the models. An extended-range investigation of the *K*β satellite may reveal closer agreement with predictions for *P*(∞) at higher incident energies.

While *P*(∞) is defined in *GRASP* as the total *n* = 2 shake-off probability in the sudden limit, experimentally it is inferred from the limiting fluorescent intensity relative to the entire *K*β spectrum (*I*_sat_/*I*_*K*β_). Additional physical mechanisms may be present that limit the utility of this comparison for *K*β. As Fig. 11 ▸ depicts, experimental satellite intensities are consistently a factor of 1.5–2 times lower in *K*β than in *K*α. As hypothesized by Melia *et al.* (2023 ▸) and Dean *et al.* (2024 ▸), this may result from differences in non-radiative Auger decay rates between single-body and shake-off decay channels in manganese *K*β, reducing the relative yield of fluorescent satellite transitions within the total *K*β spectrum.

The 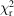 values in Table 1 ▸ indicate that the Thomas model provides an improved fit within the experimental energy range. This aligns with findings for the *K*α satellite. The parameters in Table 1 ▸ are consistent in the Thomas model for *K*α and *K*β, with the only significant deviations emerging in Δ*E* and *P*(∞). This aligns with trends for the onset and limiting intensity observed experimentally. Poor performance near the onset is evident for each of the models, exemplified by the large statistical uncertainty in Δ*E*, which totals 10%–35%, and significant deviation in Δ*E*. Statistical uncertainties are notably lower for *R* than Δ*E* for each of the models, perhaps indicating negative correlation between the two parameters, as each of the models possesses some degree of *E*_B_–*R* dependence. The models may thus compensate for larger Δ*E* with smaller *R* and *vice versa*, indicating that the radius parameter is somewhat arbitrary, hence our observations of its relatively poor consistency here.

### 6.3. Application to independent test data

Predictions made by the three literature models are prone to fitting bias when trained solely on the XR-HERFD data set, which measures only a relatively small sample of *E*_inc_ past the satellite onset. To assess the feasibility of the predicted satellite intensity (*I*_sat_) and the inferred satellite structure from PCA (*p*_1_), we require independent data at higher *E*_inc_, where the satellite intensities will ‘saturate’ and thus the structure will be fully evolved. An appropriate data set was recorded on I20-scanning, a high dwell time and low spacing HERFD-XES scan at *E*_inc_ = 9000 eV. To extract the satellite structure from the test data, we subtract the PCA-derived background [μ′, illustrated in Fig. 15(*b*) in Appendix *C*[App appc]] with appropriate normalization and alignment corrections.

**Figure 12:**
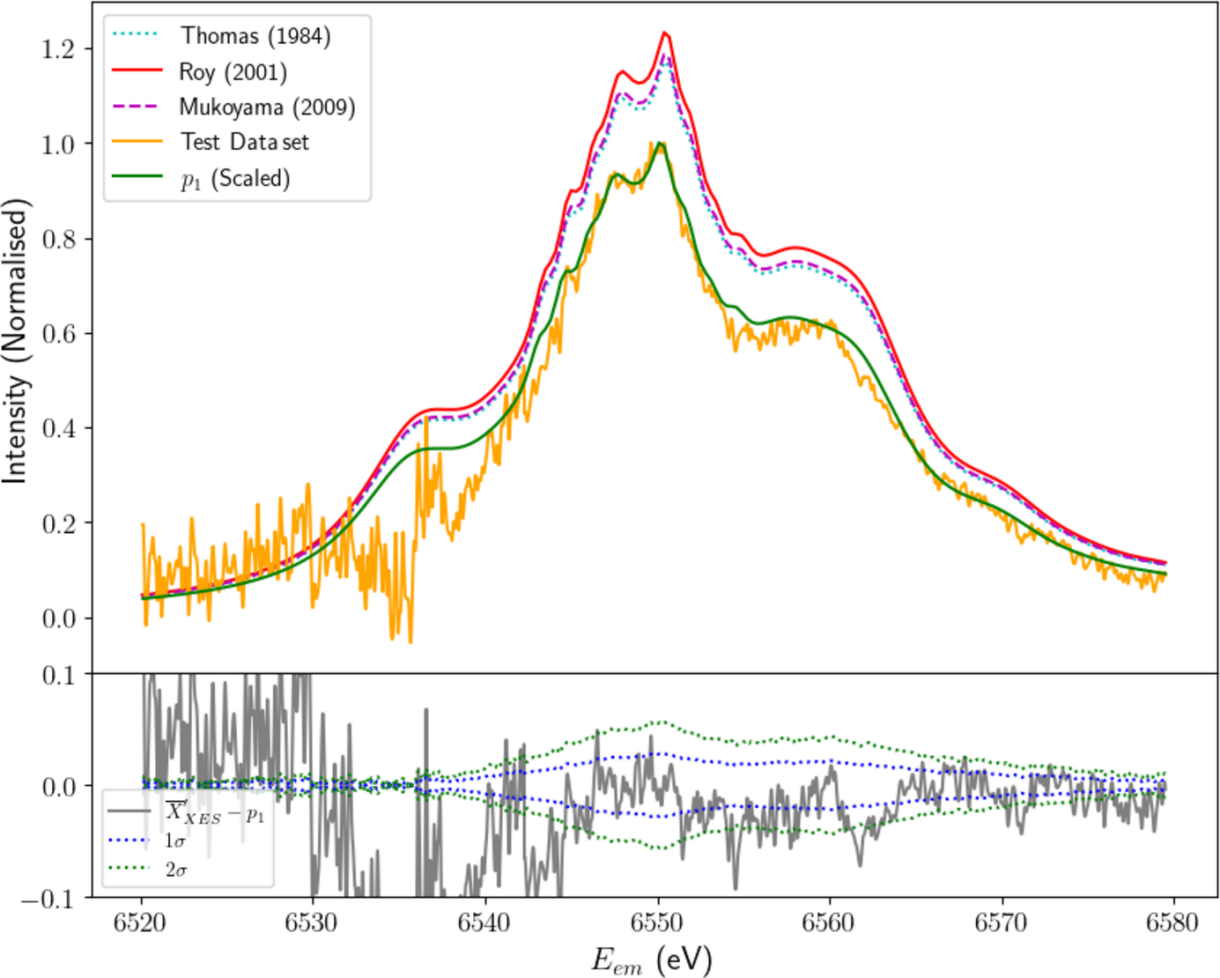
Predictions of satellite structure and intensity using the Thomas, Roy and Mukoyama models for the evolution of shake probabilities. The models are evaluated against the test data set, a background-subtracted HERFD-XES scan collected at an incident energy of 9000 eV, beyond the range of our experimental training data. Visual inspection reveals that all three models overestimate observed satellite intensities. This discrepancy may stem from intrinsic limitations of the models or, alternatively, from systematic errors or noise in the test data that reduce the measured satellite intensity. Comparison with the satellite component (*p*_1_), scaled to match the intensity of the test data, indicates excellent agreement with the structure predicted using PCA, particularly in the fine structure surrounding the main dual peak at 6550 eV and high-energy shoulder at 6560 eV. This strong consistency underscores the efficacy of PCA as a tool for probing suppressed satellite structures.

**Figure 15:**
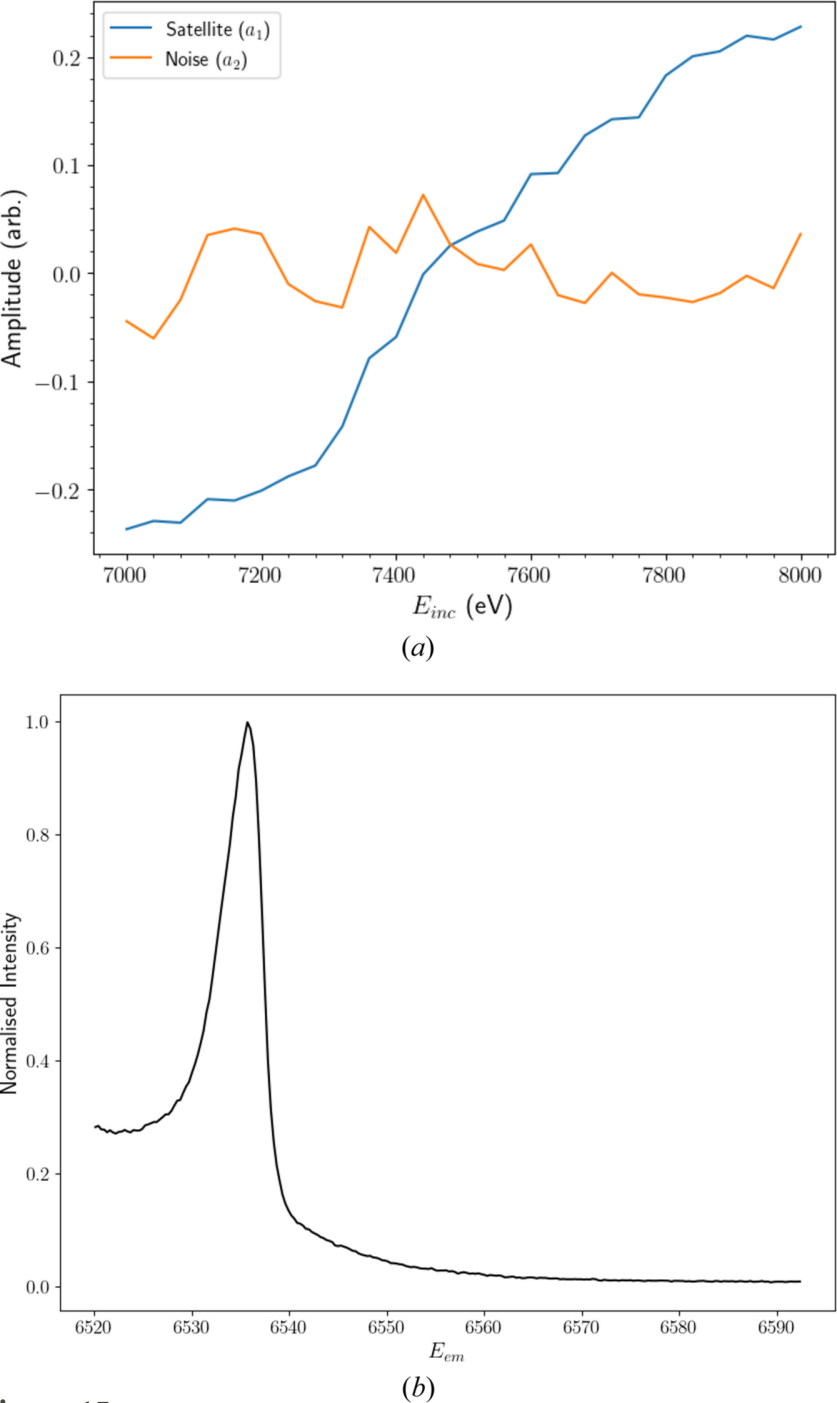
(*a*) Amplitude of the satellite and correlated noise PCs obtained without specifying a predefined mean in PCA. While the form of *a*_1_ closely resembles that shown in Fig. 9, the amplitudes are shifted, evolving from negative to positive values. This is a poor representation of the satellite’s evolution. (*b*) Stationary background profile (μ′) of the PCA data set. In the results presented in Section 5, variance across *X* was measured from μ′, as opposed to the true mean μ, to measure the evolution of satellite intensities accurately beyond the onset. μ′ constitutes a significant improvement on the background profile depicted in Fig. 2(*b*), as there is a significant reduction in noise within the satellite region.

Fig. 12 ▸ illustrates the test data and its comparison with the models. Intensities were extracted by extrapolating the intensity ratio (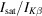) at *E*_inc_ = 9000 eV for each model from Fig. 11 ▸ to determine the predicted satellite intensity (*I*_sat_) relative to the measured *K*β intensity in the test data. All three models overestimate the measured satellite intensity by >20%. Notably, the satellite intensity in the test data is only marginally higher than it was in the high *E*_inc_ HERFD-XES slices from the training data, reinforcing our earlier observation that the *K*β satellite intensity begins to asymptote around 7800 eV. While this may highlight intrinsic limitations of the models, it would be premature to draw any definitive conclusions given the limited scope of the test data (a single XES scan). The lower-than-expected satellite intensities may stem from undetected systematic errors or noise which could contribute to errors of >10% in the measured intensity ratio.

The structural agreement between the PCA model and the test data is confirmed, demonstrated by the scaled PCA satellite profile (from Fig. 8 ▸). The test data exhibit the same dual-pronged main peak and high-energy shoulder, with fine structure in these regions aligning closely. Comparing the two structures above *E*_em_ = 6540 eV, where systematic errors are minimized, yields a 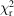 of 7.81, indicating strong agreement, especially given the low data uncertainty in the high-statistics test data set.

Below 6540 eV, significant deviations emerge. The low-energy shoulder appears sharper and more distinctly separated from the main satellite peak than predicted by the training data. This discrepancy may result from noise artefacts or misalignment causing a shift in the Kβ_2,5_ peak location. Such behaviour is not unprecedented, as a similar sharp dip in intensity is visible in the PCA satellite component in Fig. 7 ▸(*a*). While an initial analysis of energy-eigenvalue calculations for the *K*β satellite reported by Dean *et al.* (2024 ▸) supports the presence of the low-energy shoulder, the scope of our data leaves it unclear whether it originates from a real physical phenomenon or merely from fluctuations in noise that have contaminated the first PC.

## 7. Conclusion

This work presents XR-HERFD measurements of the manganese *K*β spectrum across an extended range of *E*_inc_, enabling the discovery of a new satellite in the high-energy tail of *K*β_2,5_. This marks the first observation of such a satellite in *K*β spectra. The high precision of these results allowed the satellite to be resolved with a standard error signature of 35 σ_se_ per pixel along the satellite peak and 652 σ_se_ across the full spectrum, far exceeding conventional discovery thresholds. This underscores the value of XR-HERFD in detecting new or known physical processes.

Application of principal component analysis (PCA) extracted detailed structural information on the satellite, depicting fine structure that suggests multiple physical origins, as is typical of shake processes. The significance of the satellite was further underscored by its contribution to over 95% of variance from the *K*β background, with correlated noise accounting for less than 5%. Given the onset and structure of the satellite, we attribute its physical origin to *n* = 2 (2*s* and 2*p*) shake-off events following core photoionization, producing a spectrum non-degenerate to the characteristic *K*β_1,3_ when a 3*p* electron fills the core hole.

Regression analysis following PCA allowed scaling parameters for the satellite (α_1_) and *K*β background (β_1_) to be determined at each *E*_inc_ in the experimental energy range, and thus the relative intensity 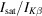 could be defined. This provided a highly accurate depiction of the satellite’s evolution, the first such example for *K*β spectra.

Near-degeneracy and low intensities near the onset complicate the separation of individual shake components. Consequently, the satellite and its evolution were considered through a single component. Future work will focus on resolving the physical components of the satellite fine structure through a detailed statistical analysis using theoretical models, allowing for the extraction of the independent evolution of each satellite component.

Fitting literature models to the evolutionary data enabled quantification of key physical parameters. As was the case for the *K*α satellite, the Thomas model yielded the lowest 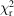 of 3.46. The presence of additional physical mechanisms such as Auger suppression in the satellite channel may affect the consistency of the process amplitude between *K*β and *K*α, warranting further investigation through *ab initio* calculations.

Further evidence for the evolving intensities in shake-off processes reinforces the need to refine the many-body reduction factor 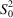 as a varying function of incident photon energy. While the *n* = 2 satellite has a limiting contribution to overall fluorescent intensities in manganese, shake-off processes with onsets closer to the absorption edge may contribute upwards of 20–30% (Dean *et al.*, 2024 ▸; Nguyen *et al.*, 2022 ▸), providing a significant energy dependency to 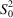. A fully comprehensive representation of 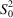 should incorporate the evolution of all many-body processes in a given material and continue to decrease past the absorption edge. Such a framework will provide deeper insights into many-body interactions and facilitate significant improvements in the extraction of structural parameters in condensed-matter materials from XAFS measurements.

## Figures and Tables

**Figure 13:**
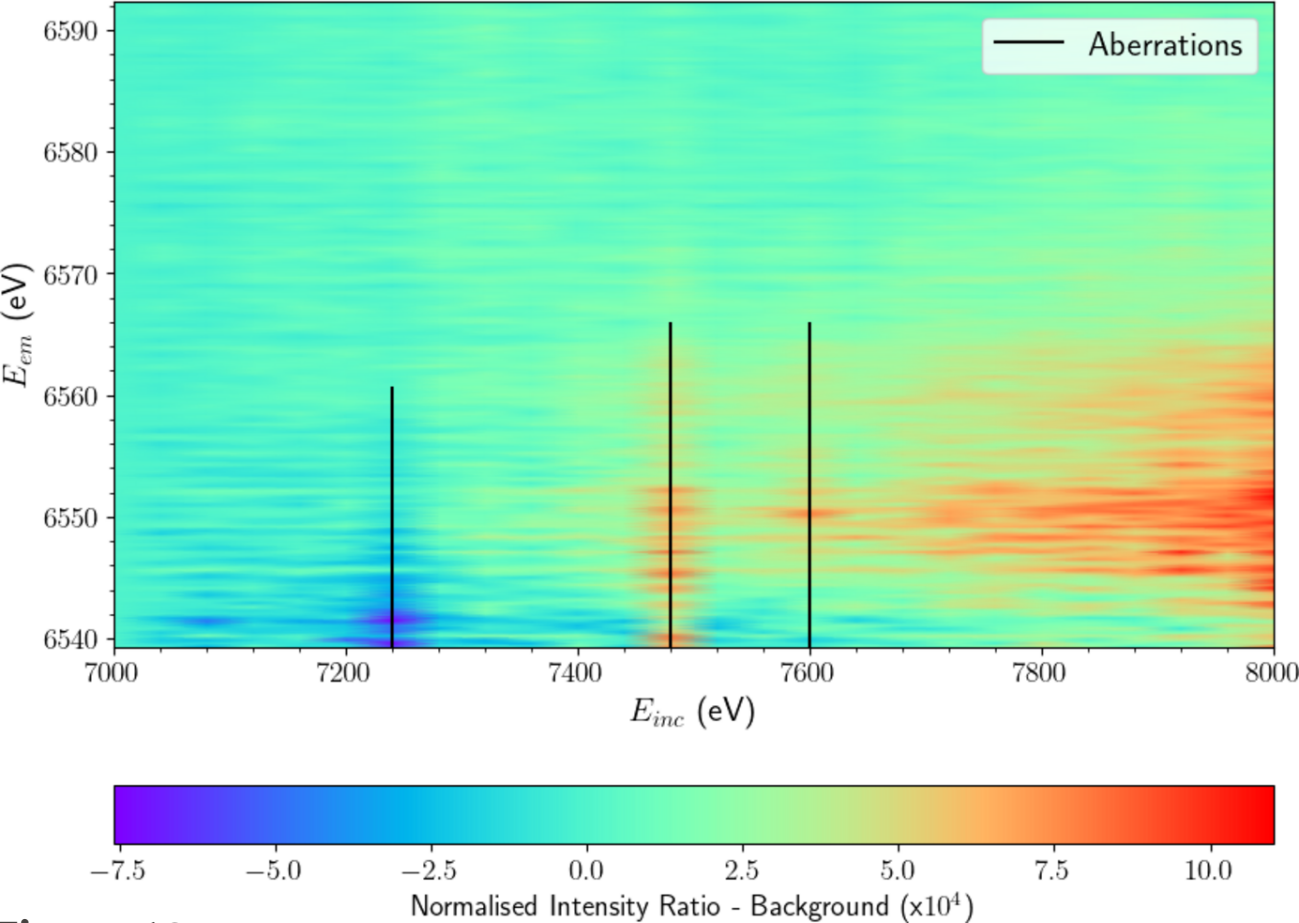
Uncorrected background-subtracted intensities using 14 analyser crystals. Vertical intensity spikes are evident, corresponding to fluctuations in the HERFD-XES slices at incident energies *E*_inc_ = 7240, 7480 and 7600 eV. These are best attributed to electronic noise spikes in the Medipix detectors and significantly decrease the signal-to-noise ratio of the satellite. To correct for this, affected slices are replaced by the average of the surrounding data points. The improvements can be seen in Fig. 14.

**Figure 14:**
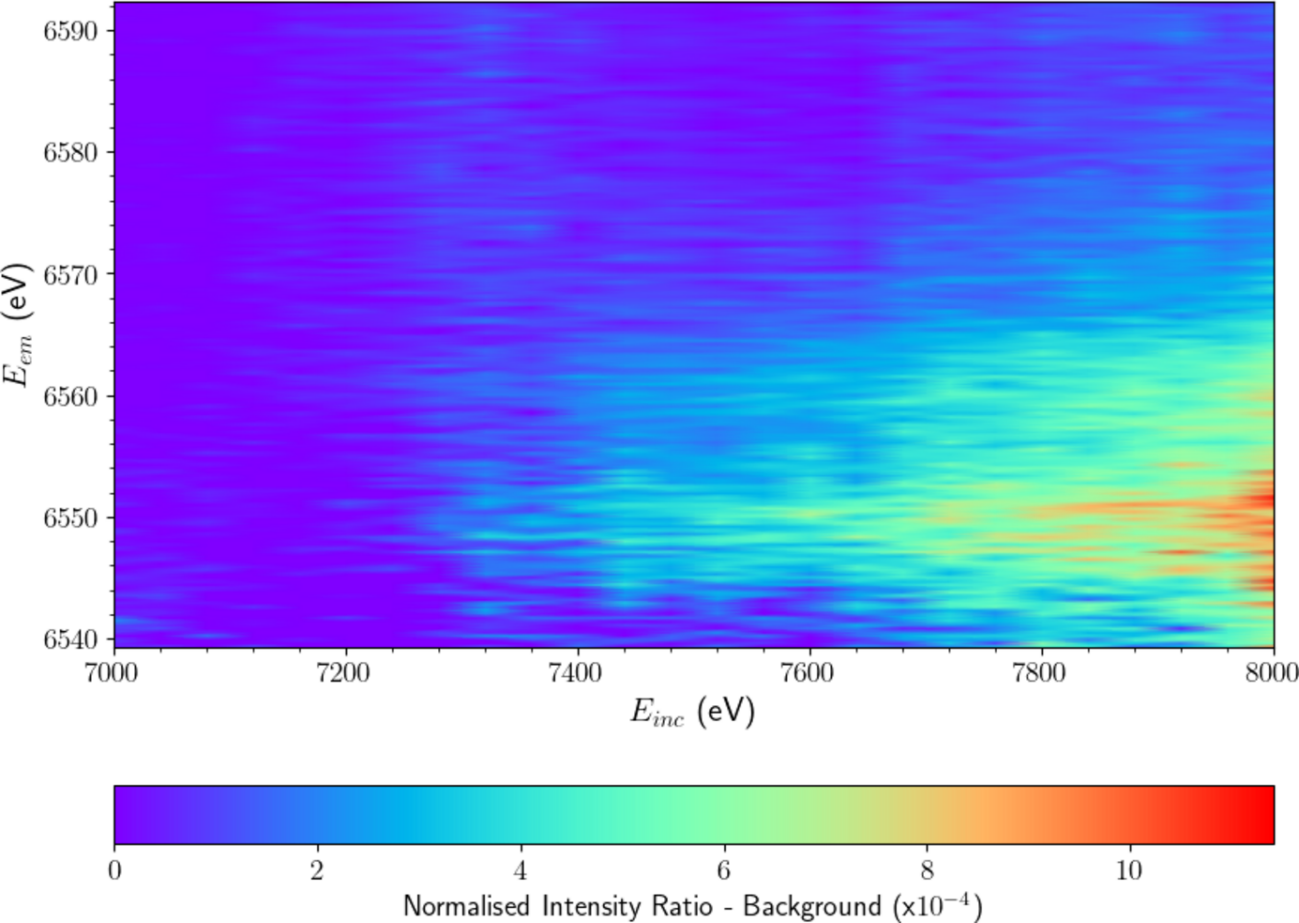
XR-HERFD map after subtraction of the *K*β background using all 14 analyser crystals. The satellite structure and evolution are difficult to resolve. A closer analysis of the fluorescent intensities from each analyser crystal revealed that six of the analysers delivered a signal of poor statistical quality, significantly increasing the signal-to-noise ratio. These six crystals where excluded from the final processed data seen in Fig. 5, resulting in a much clearer image of the satellite’s structure and evolution.

**Table 1:** Fitting parameters and associated statistical uncertainties extracted from the Roy, Thomas and Mukoyama models for the evolution of the *K*β satellite *Ab initio* calculations alongside fitted parameters for the Thomas model (determined the best performing) for the *K*α satellite in Sier *et al.* (2024 ▸) are included as a reference point. *P*(∞) is the limiting shake probability relative to the entire *K*β spectrum in the sudden limit, *R* is the radius of the 2*p* (shake) orbital and Δ*E* is the difference in 2*p* binding energy relative to the computed value in *GRASP*.

Model	Thomas (1984 ▸)	Roy *et al.* (2001 ▸)	Mukoyama *et al.* (2009 ▸)	Sier *et al.* (2024 ▸)	Thomas (1984 ▸) (*K*α)
*P*(∞) (%)	0.60 ± 0.052	0.85 ± 0.086	0.66 ± 0.12	1.11	1.041
*R* (Å)	0.105 ± 0.0076	0.129 ± 0.016	0.131 ± 0.021	0.098	0.1074
Δ*E* (eV)	−104.9 ± 16.2	−53.5 ± 5.08	−19.9 ± 7.11	0	−62.13
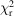	3.46	3.94	6.55	–	3.39

## Data Availability

The data supporting the findings of this study are available upon request.

## Appendix A Removal of intensity aberrations

As outlined in Section 4, the satellite intensities in *K*β are several orders of magnitude lower than the spectrum and so even minor fluctuations in detector counts can significantly reduce the signal-to-noise ratio. Fig. 13 ▸ illustrates this issue, displaying several vertical intensity spikes that correspond to fluctuations in three individual HERFD-XES slices within the XR-HERFD array. These spikes are unlikely to originate from physical processes and are instead better attributed to false counts in the Medipix detectors caused by electronic noise.

To ensure the satellite is well resolved, these aberrations are removed from the final data and replaced by the average intensity of adjacent data points. For instance, in the affected HERFD-XES scan at *E*_inc_ = 7240 eV, the fluorescent intensity at each *E*_em_ is replaced by the average of the corresponding intensities at *E*_inc_ = 7200 and 7280 eV. While this correction alters the raw experimental data, its primary purpose is to enhance the statistical accuracy of the satellite measurement. The effectiveness of this approach is demonstrated by a comparison of Figs. 13 ▸ and 14 ▸.

## Appendix B Selection of analyser crystals

As the experimental apparatus in Fig. 1 ▸ depicts, the 14 analyser crystals are arranged such that their fluorescence signals are completely separable on the two detector apparatuses, allowing the output from each crystal to be analysed independently. As discussed in Section 4, this allowed us to identify that six of the 14 crystals produced fluorescence data with significantly lower statistical quality, to the point that their inclusion actively reduced the signal-to-noise ratio of the overall data set. To rectify this, fluorescent counts from only the eight crystals with the highest statistical quality were included in the final data. A comparison of Fig. 14 ▸, which uses all 14 crystals, and Fig. 5 ▸, which uses only eight, clearly demonstrates that this processing step significantly improves the resolution of the satellite region’s structure and evolution.

The cause of the statistical inconsistencies between the 14 analyser crystals is difficult to determine precisely, but is most likely due to a combination of systematic errors including misalignment, calibration issues or non-uniform analyser curvature. These inconsistencies stem largely from the pioneering experimental setup, as the 14-analyser apparatus had been implemented only a few months prior to our experimental run time on I20-Scanning. Recent upgrades have rectified these issues, ensuring all 14 analysers consistently deliver high-quality data, significantly enhancing the resolving power of future investigations.

Our ability to resolve the satellite despite these systematic issues highlights the advantages of independently projecting signals from each analyser crystal. If intensities had instead been homogenized onto a single region of the photon detector array, any faults in an individual analyser would have introduced irreversible distortions into the data set. Furthermore, failing to separate analyser signals would hinder the effective application of the splicing methodology, leaving experimental results vulnerable to spectral broadening and misalignment artefacts.

## Appendix C Principal component analysis in XR-HERFD

The application of PCA discussed in Section 5 uses a singular value decomposition algorithm (SVD) (rather than a least-squares or Bayesian analogue). SVD-based PCA applies to many fields in the current era of big data (Wang & Zhu, 2017 ▸), including geochemistry (Praus, 2005*a* ▸), gene expression (Wall *et al.*, 2003 ▸), facial recognition (Asiedu *et al.*, 2016 ▸) and hydrology (Praus, 2005*b* ▸). Using SVD, any real *n*×*m* data matrix *Y* of rank *r* [] can be factorized as *Y* = *BSC*^T^ (Wang & Zhu, 2017 ▸). *S* is a diagonal matrix whose entries, the ‘singular values’, correspond to the eigenvalues of *YY*^T^ and are ordered by decreasing magnitude (Kim & You, 2023 ▸).

In SVD-based PCA, the singular values correspond to the ordered eigenvalues of the covariance matrix Σ, obtained by performing SVD on the mean-centred data:  = , where μ is the mean of the data set. Since Tr(Σ) is representative of the total variance across *Y*, the magnitude of each eigenvalue is indicative of the proportion of the total variance represented by the corresponding PC.

Our XR-HERFD data can be interpreted as an  matrix *X* of rank , where  and  are the numbers of incident energies and emission energies, respectively, in the XR-HERFD data set.

Unlike in standard applications of PCA, we wish to isolate the key components of variance from the stationary background profile, as opposed to the mean μ of the HERFD-XES slices in *X*, which incorporates not only the *K*β background but also the satellite.

Application of the PCA algorithm using the uncorrected mean μ is depicted in Fig. 15 ▸(*a*). The amplitude of the satellite component increases from negative to positive, behaviour inconsistent with the expected characteristics of an evolutionary profile.

To improve this and measure the appropriate variance, we modified the PCA functionality to allow the subtraction of a pre-defined mean before extracting the PCs. This mean was determined by correcting μ using the minimum amplitude *a*_1,min_ of the satellite component in Fig. 15 ▸(*a*). This allows us to extract the refined background profile μ′ by subtracting the PC satellite (*p*_1_) weighted by ,

This ensures that the contribution of the satellite to the background is 0. The resulting form of μ′ is depicted in Fig. 15 ▸(*b*) and corresponds to the form of the *K*β_1,3_ and *K*β_2,5_ background profiles bereft of any satellite contribution. This process ensures a significant reduction in noise in the high-*E*_em_ region of μ′, where we expect to find the satellite, compared with the background we extracted by averaging across relevant HERFD-XES slices [Fig. 2 ▸(*b*)].

We then perform SVD on the refined background-subtracted data  by

The columns of *P*, the right singular values *p*_*i*_, represent the loadings (which we refer to as ‘weights’ in Section 5) of each of the emission energies in the *i*th principal component. The entry *p*_*i*,*k*_ is indicative of the degree to which intensities at the *k*th emission energy vary across the *i*th PC axis. The loadings of the first two PCs are discussed in detail in Section 5 (Fig. 7 ▸). After correction of the mean, the structure of *p*_1_, the satellite loadings, remains largely unchanged yet accounts for 10% more variance across the XR-HERFD data set as a result of the improved accuracy of the reference point.

The columns of *A* (the left singular values *a*_*i*_) each represent one of the PCs and are equivalent to a linear combination of the entries in each of the background-subtracted HERFD-XES slices in  weighted by the PC loadings, . The entries of *a*_*i*_ (*a*_*i*,*j*_) thus represent the ‘score’ of the *j*th HERFD-XES slice in the *i*th PC.

We refer to the scores as ‘amplitudes’ in Section 5. Upon correction of the background, the scores of the satellite component (*a*_1_) correspond to the appropriate form for the satellite evolution profile (Fig. 9 ▸).
